# Physico-chemical properties and substrate specificity of α-(1→3)-d-glucan degrading recombinant mutanase from *Trichoderma harzianum* expressed in *Penicillium verruculosum*

**DOI:** 10.1128/aem.00226-24

**Published:** 2025-01-23

**Authors:** Olga A. Sinitsyna, Pavel V. Volkov, Ivan N. Zorov, Alexandra M. Rozhkova, Oleg V. Emshanov, Yulia M. Romanova, Bozhena S. Komarova, Natalia S. Novikova, Nikolay E. Nifantiev, Arkady P. Sinitsyn

**Affiliations:** 1 Department of Chemistry, M.V. Lomonosov Moscow State University 204326 , Moscow, Russia; 2 Federal Research Centre «Fundamentals of Biotechnology» of the Russian Academy of Sciences 442108 , Moscow, Russia; 3 LLC BFR Laboratories, Moscow, Russia; 4 The National Research Center for Epidemiology and Microbiology named after Honorary Academician N.F. Gamaleya of the Ministry of Health of the Russian Federation, Moscow, Russia; 5 Laboratory of Glycoconjugate Chemistry, N.D. Zelinsky Institute of Organic Chemistry, Russian Academy of Sciences 54744 , Moscow, Russia; Washington University in St. Louis, St. Louis, Missouri, USA

**Keywords:** mutanase, α-1,3-glucanase, α-(1→3)-d-glucan, oligo-α-(1→3)-D-glucoside substrates, *Penicillium verruculosum*, bacterial biofilms

## Abstract

The gene *mutAW* encoding *Trichoderma harzianum* fungus mutanase (MutA, GH71 family, α-1,3-glucanase, EC 3.2.1.59) was cloned and heterologously expressed by the highly productive *Penicillium verruculosum* fungus. *P. verruculosum MutA* strain secreted crude enzyme preparations with the recombinant MutA content of 40% of the total secreted protein, and the specific activity increased 150 folds compared to that of enzyme preparation obtained by the host strain. Homogeneous MutA had molecular mass of 70 kDa and displayed maximum of the activity on mutan at pH 5.0 and 50°C, with *K*_m_ and *k*_cat_ being 1.0 g/L and 30 s^−1^, respectively. At 40-50°C, the MutA was stable for at least 3 h. Glucose was the main product of long-term mutan hydrolysis. HPLC analysis of hydrolysis product of oligo-α-(1→3)-D-glucosides bearing UV-detectable *N*-*trans*-cinnamoyl residue in the aglycon clearly indicated that MutA has an endo-processive hydrolytic mode of action. It was demonstrated that MutA can destroy the polysaccharide matrix of both gram-positive and gram-negative pathogenic bacteria biofilms.

**IMPORTANCE:**

The manuscript describes the properties of a novel recombinant GH71 mutanase Mut A from *Trichoderma harzianum*. Gene *mutAW* encoding mutanase was heterologously expressed in the host strain *Penicillium verruculosum* B1-537 (ΔniaD). The recipient strain has a high secretory ability and allowed to obtain preparations containing the target recombinant enzyme up to 80% of the total protein pool. MutA exhibited a high activity against mutan and negligible or zero activity toward other types of glucans including α-(1→4)-, β-(1→3)-, β-(1→4)-, and β-(1→6)-glucans. By using a series of synthetic oligo-α-(1→3)-D-glucosides, we demonstrated that MutA is an endo-processive enzyme, which hydrolyzes the internal glucosidic bonds and releases glucose from the reducing end sliding into the non-reducing end. MutA recognizes tetrasaccharide as a minimal substrate and hydrolyzes it to trisaccharide and glucose. The effectiveness of the use of MutA for the destruction of clinical isolates of gram-positive and gram-negative bacteria is also described.

## INTRODUCTION

Mutanase (α-1,3-glucanase, EC 3.2.1.59) is the enzyme that catalyzes the hydrolysis of α-(1→3)-glucosidic linkages in mutan giving the low-molecular-weight products. Mutan is a major component of exopolysaccharides produced by bacteria like tooth-colonizing streptococci such as *Streptococcus mutans* (1, 2), pathogenic fungi like *Aspergillus fumigatus* (3), *Fusarium oxysporum* or *Botrytis cinerea* (4), and other species containing α-(1→3)-d-glucans in their cell wall. It is composed of the main α-(1→3)-glucan chain with some α-(1→6)-branches. Mutans are tenacious, water insoluble and alkali soluble; in contrast to other matrix components, they are not attacked by enzymes present in the plaque. Mutanase has been tested for its potential as a caries-preventive agent due to its ability to remove biofilms created by oral bacteria *in vitro* (5) and to reduce plaque formation *in vivo* (6). Moreover, applications of mutanase are related to their antifungal effect against phytopathogenic fungi containing α-(1→3)-glucans in their cell wall, like *Fusarium oxysporum* or *Botrytis cinerea* (4).

Mutanases are produced by bacteria (7, 8) and filamentous fungi (9–11). The latter group, predominantly consisting of *Trichoderma* species, represents the most extensively studied and reviewed sources of α-(1,3)-glucanases (12–14). According to the CAZy database (http://www.cazy.org/), practically all known mutanases are classified into families 71 and 87 of glycoside hydrolases (GH71 and GH87). Bacterial mutanases are present mostly in the GH87 family, while those from fungi belong exclusively to the GH71 family.

In this paper, we describe the properties of recombinant GH71 mutanase from *T. harzianum*. Gene *mutAW* encoding mutanase was heterologously expressed in the host strain *Penicillium verruculosum* B1-537 (Δ*niaD*). The recipient strain has a high secretory ability (up to 50–60 g of extracellular protein per 1 L of fermentation broth), and this strain has been successfully used for the production of different homologous and heterologous enzymes and allowed to obtain preparations containing the target recombinant enzyme up to 80% of the total protein pool (15–17).

## MATERIALS AND METHODS

### Microbial strain

The mycelium of the *T. harzianum* strain, obtained from the surface of a crumbling tree (this strain is identical to the strain from the VKPM collection F-114), was used for isolation of the genomic DNA with DNeasy Plant Mini Kit (QIAGEN, Valencia, CA). The identity of the *Trichoderma harzianum* strains was confirmed based on the match of the 28S, *tef1* and *rpb2* barcode sequences in accordance with the methodology described by Cai et al. (18). Auxotrophic *P. verruculosum* B1-537 strain, deficient in the nitrate reductase gene (Δ*niaD*), was used as host strain for transformation and chromosomal DNA preparation. The *Escherichia coli* MachIT1 strain (Thermo Fisher Scientific, Waltman, MA) was applied for bacterial transformation and isolation of plasmids containing target genes. *Aspergillus niger* ATCC 10864 strain was used for mutan production.

### Protoplast preparation

The *P. verruculosum* mycelium was grown on minimal medium (MM) at 32°C for 6 days. Mycelium from plate harvested from the MM plate and transferred into 100 mL of liquid MM, containing 10 mM NH_4_Cl and incubated at 28°C, 180 rpm for 4 days. On the fourth day, *P. verruculosum* mycelium was harvested by filtering through miracloth and rinsed two times with protoplasting buffer (1.2 M MgSO_4_, 10 mM NaH_2_PO_4_, pH 5.0). The mycelium (approximately 2.0 g fresh weight) was then transferred into a 15 mL glass tube. Exactly 4 mL of filtered sterile lysis enzyme solution [3% of lysing enzymes from *T. harzianum* (Sigma-Aldrich, USA) dissolved in PB] was added to the *P. verruculosum* mycelium. The mycelium-enzyme mixture was incubated at 30°C, 150 rpm for 3 h. About 10 mL of 0.6 M MM buffer was added into the mycelium-enzyme mixture to dilute the concentration of enzyme. The *P. verruculosum* protoplasts were separated from cell wall debris using a glass filter. Centrifugation was carried out at 1,575 × *g* for 10 min to collect the protoplasts. The supernatant was carefully decanted, and about 10 mL of 0.6 M MM buffer was added to wash the protoplast pellet. The tube was inverted several times carefully and centrifuged at 1,575 × *g* for 20 min. The supernatant was decanted, and the *P. verruculosum* protoplasts were resuspended in 100 µL of SCT buffer (0.6 M sorbitol, 10 mM Tris/HCL, and 10 mM of CaCl_2_, pH 7.5).

### Construction of the expression plasmids and transformation of *P. verruculosum* B1-537 (ΔniaD) strain

Genetic constructions and production of recombinant strains were carried out as described by Sinitsyn et al. (19). Briefly, using the freshly isolated genomic DNA of *T. harzianum* fungus and two pairs of primers shown below, the ~1,900 bp PCR-product was amplified by polymerase chain reaction (PCR) using Bio-Rad T1000 thermal cycler (ThermoFisher Scientific, Waltham, MA) for further cloning into *P. verruculosum* B1-537 host strain:

Mut_Thar-LIC5—caaacagaagcaaccgacacaatgttgggcgttttccgccgcctcag

Mut_Thar-LIC3—gaggagaagcccggttagcagtattgacatgccgttggggggcag

Then, using the method of independent ligation (LIC-cloning method) (20), the *mutAW* gene was cloned into the pCBHI vector in the same manner as it was previously described (21). As a result, the plasmid pCBHI-Mut was obtained (Fig. S1). The protoplast of *P. verruculosum* B1-537 recipient strain was transformed by the pCBHI-Mut plasmid together with the co-transforming plasmid pSTA10 at the ratio of 6:1 µg/µL. For one transformation experiment, we used 3*10^7^ quantities of protoplasts. Ten micrograms of target and 1 µg of co-transformation (pSTA10) plasmids were mixed, after that mixed with 100 µL of protoplast solution, incubated for 20 min in the cold, and then 500 µL of PCT buffer (50% PEG-6000, 10 mM CaCl_2_, 10 mM Tris/HCl, pH 7.5) was added. Centrifugation was performed at 1,500 *g* for 15 min. The resulting precipitate protoplasts were redissolved in 200 µL of 1 M sorbitol and seeded on agarized dishes with a minimal medium containing 1 M sorbitol. The pSTA10 plasmid contained a nitrate reductase (*niaD*) gene, allowing the selection of the resulting transformants on medium with sodium nitrate (NaNO_3_, 10 mM). An indicator of the effective transformation of the *P. verruculosum* B1-537 was the production of 20–40 clones per 1 µg of pCBHI-Mut plasmid, which corresponds to the literature data for *Penicillium sp.* strains (22). The absence of additional mutations, deletions, or insertions in the *mutAW* gene was confirmed by its sequencing in both directions according to Sanger et al. (23).

### Cultivation of strains and screening of transformants

*T. harzianum* strain was cultivated in shake flasks (total volume 750 mL, fermentation medium volume 100 mL) during 144 h at 30 ± 0.1 °С on a medium containing (in g/L): glucose—5.0, yeast extract—10.0, potassium phosphate—25.0.

Screening of transformants was carried out in shake flasks (total volume 750 mL fermentation medium volume 100 mL). A medium for cultivation of *P. verruculosum* transformants had the following composition (in g/L): glucose—40; microcrystalline cellulose (MCC)—40; wheat bran—10; corn extract—30; КН_2_РО_4_—14; (NH_4_)_2_SO_4_—1; MgSO_4_·7H_2_O—0.6; СaCl_2_·2H2O—0.6 (рН 5.0 ± 0.25, 32 ± 0.1 °С, fermentation time 144 h). The recipient strain of *P. verruculosum* B1-537 (Δ*niaD*) was used as a control. The best clones selected were cultivated in 3 L fermenters KF-104/3 (Prointech LLC, Puchino, Russia) using the same fermentation medium as above. Fermentation was carried out in fed-batch mode with fractional glucose addition (every 24 h after the first 48 h) and three additions of MСС (every 24 h after the first 72 h). At the end of the cultivation process, the fermentation broth was centrifuged (6,500 rpm, 40 min) at Avanti JXN-26 centrifuge (Beckman Coulter, Brea, CA) to remove fungal biomass and insoluble components of the nutrient medium. The supernatant was freeze-dried using VirTis BenchTop 2K ES freeze dryer (SP Scientific, Gardiner, NY).

*A. niger* 10864 ATCC strain was cultivated in a shake flask (total volume 3 L, fermentation medium volume 1.5 L, 250–600 rpm) for 144 h at 30 ± 0.1°С on a medium containing flour mash treated with amylase. Crude biomass yield was 850 g.

### Enzyme purification

Desalting and fractionation of crude freeze-dried enzyme preparations were carried out on ACTA Purifier system (GE Healthcare, UK) by anion-exchange chromatography (AEC) on a Source 15Q column (Cytiva, Sweden), followed by hydrophobic interaction chromatography (HIC) with a Source 15ISO column (Cytiva, Sweden) and gel filtration on Superose 12 column (GE Healthcare, Sweden) like it was described in reference 21. The enzyme purity was characterized by sodium dodecyl sulfate polyacrylamide gel electrophoresis (SDS-PAGE). SDS-PAGE was carried out in 12% gel using Mini Protean Tetra cell, and isoelectrofocusing was made with Model 111 Mini IEF cell (Bio-Rad Laboratories, USA). Staining of protein bands was carried out in Coomassie Blue R-250 (Ferak, Germany). Protein concentration in samples was determined by the modified method of Lowry et al. (24), using bovine serum albumin as the standard.

### Enzyme identification by MALDI-TOF mass spectrometry

Identification of mutanase was carried out by MALDI-TOF mass spectrometry (MS) peptide fingerprinting of trypsin-digested proteins from the bands after SDS-PAGE as described elsewhere (25). MALDI-TOF MS of peptides was carried out on an UltrafleXtreme TOF/TOF mass spectrometer (Bruker Daltonik GmbH, Bremen, Germany). Peptides in the mass spectra were identified using the online service FindPept (https://web.expasy.org/findpept/) with translated from the *mutA* gene amino acid sequence of *T. harzianum* mutanase. They were compared with the peptides obtained by theoretical trypsinolysis of the *mutA* gene amino acid sequence of *T. harzianum* mutanase.

### Isolation of α-(1→3)-d-glucan (mutan) from *A. niger* biomass

The *A. niger* fungus was fermented in a 3 L fermenter on a medium containing α-amylase treated wheat flour as a carbon source. Isolation of α-(1→3)-d-glucan (mutan) from *A. niger* biomass was carried out according to the method described by Wiater et al. (26). The resulting biomass was centrifuged at 4,000 rpm, filtered, and squeezed. The obtained substance was washed twice with distilled water, squeezed again; pH was adjusted to 7.0 with 1 M HCl. The suspension was centrifuged for 15 min at 7,000 rpm and lyophilized. The dried material (100 g) was successively extracted with methanol, NaCl (9 g/L), hot water, Na_2_CO_3_ solution (50 g/L), and, finally, 1 M NaOH containing 0.2 g/L NaBH_4_ for 24 h at room temperature. The alkali extract was neutralized with 1 M HCl. The precipitated polysaccharide fraction was washed several times with water, collected by centrifugation, and lyophilized.

### Synthesis of *N*-trans-cinnamoylated oligo-α-(1→3)-D-glucoside probes

**Fig 1:**
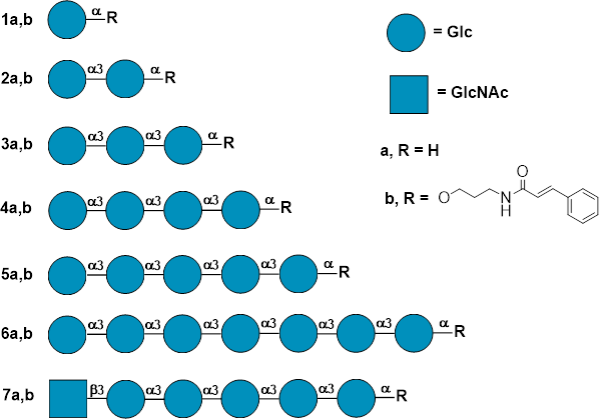
The structures of synthetic *N*-*trans*-cinnamoylated glycoconjugates **1b–7b** studied as mutanase substrates and of parent oligosaccharide aminopropyl glycosides **1a–7a**. The carbohydrate sequences are represented according to symbol carbohydrate nomenclature (30).

The synthesis of *N*-cinnamoylated oligo-α-(1→3)-D-glucoside probes **1b-7b** (Fig. 1) was performed according to the typical procedure exemplified for the preparation of 3-*N*-*trans*-cinnamidopropyl α-d-glucopyranoside **1b** from parent oligosaccharide aminopropyl glycosides which were described before: **4a** and **7a** in reference 27, **2a** and **3a** in reference 28, and **5a** and **6a** in reference 29. The cinnamic acid aglycone was chosen because of its small size, which minimizes interference with enzyme activity and solubility issues, combined with a relatively high extinction coefficient (*ε* ≈ 10,000–15,000 M⁻¹·cm⁻¹) that enables the detection of low concentrations of tagged samples using standard fluorescent detection equipment.

In order to obtain 3-*N*-*trans*-cinnamidopropyl α-d-glucopyranoside (**1b**, Fig. S2) Et_3_N (2 µL) and *N*-succinimidyl *trans*-cinnamon (6.8 mg, 0.028 mmol) were added to the solution of previously desalted (with Ambersep 900 resin) monosaccharide **1b** (3.3 mg, 0.014 mmol) in absolute dimethylformamide (DMF, 295 µL) and H_2_O (29 µL), making sure that cinnamon ester is dissolved. A day later, the solvent was evaporated; the dry residue was co-evaporated with toluene three times. Compound **1b** (2.9 mg, 58%) was isolated after the column chromatography on silica gel (16–25 μm, 7:1→5.5:1).

3-*N*-*trans*-cinnamidopropyl α-d-glucopyranosyl-(1→3)-α-d-glucopyranoside (**2b**, Fig. S2) was obtained according to the typical procedure in a yield of 63% and isolated by chromatography on a TSK-40 gel column. 3-*N*-*trans*-cinnamidopropyl α-d-glucopyranosyl-(1→3)-α-d-glucopyranosyl-(1→3)-α-d-glucopyranoside (**3b**, Fig. S2), 3-*N*-*trans*-cinnamidopropyl α-d-glucopyranosyl-(1→3)-α-d-glucopyranosyl-(1→3)-α-d-glucopyranosyl-(1→3)-α-d-glucopyranoside (**4b**, Fig. S2), 3-*N*-*trans*-cinnamidopropyl α-d-glucopyranosyl-(1→3)-α-d-glucopyranosyl-(1→3)-α-d-glucopyranosyl-(1→3)-α-d-glucopyranosyl-(1→3)-α-d-glucopyranoside (**5b**, Fig. S2), 3-*N*-*trans*-cinnamidopropyl α-D-glucopyranosyl-(1→3)-α-d-glucopyranosyl-(1→3)-α-d-glucopyranosyl-(1→3)-α-d-glucopyranosyl-(1→3)-α-d-glucopyranosyl-(1→3)-α-d-glucopyranosyl-(1→3)-α-d-glucopyranoside (**6b**, Fig. S2), and 3-*N*-*trans*-cinnamidopropyl 2-deoxy-2-acetamido-β-d-glucopyranosyl-(1→3)-α-d-glucopyranosyl-(1→3)-α-d-glucopyranosyl-(1→3)-α-d-glucopyranosyl-(1→3)-α-d-glucopyranosyl-(1→3)-α-d-glucopyranoside (**7b**, Fig. S2) were obtained according to the typical procedure in a yield of 73%, 72%, 85%, 42%, and 84%, respectively, and were isolated by chromatography on a reversed phase HPLC column (Supelcosil LC-18-DB semi-prep, 25 cm × 10 mm, 5 µm, in MeOH–H_2_O; 1:1, 35:65, 32:68, 31:69, and 23:77 for 3b, 4b, 5b, 6b, and 7b, respectively).

### Enzyme activity assays

Enzyme activity against polysaccharide substrates was assayed using the modified Nelson-Somogyi method (31) of determination of reducing sugars (RS) released from mutan (from *A. niger*); pachyman and barley β-glucan (Megazyme); pustulan (InVivoGen, France); laminarin from *Laminaria digitata*, xylan from beech wood, MCC (Avicel PH-101), Na-salt carboximethylcellulose (CMC), starch from potato and dextran (Sigma, St. Louis, MO, USA) like it was described in reference 21. Enzyme activities were expressed in international units, one unit of activity corresponded to the quantity of enzyme hydrolyzing 1 µmol of substrate or releasing 1 µmol of RS per minute. All activity assays were performed in triplicates.

Enzyme activities against synthetic low-molecular-weight substrate *p-*nitrophenyl-β-D-glucopyranoside (PNPG) were assayed via the initial rates of formation of the colored reaction product *p*-nitrophenolate at pH 5.0 and 40°C. Study of effect of pH and temperature on the mutanase activity was performed using mutan as a substrate. The activity assays were carried out as described above, except pH-dependance: 0.1 M citrate/phosphate universal buffer was used for maintaining the necessary pH in the reaction system instead of the acetate buffer.

### Determination of kinetic parameters

The kinetic parameters of purified mutanase (MutA) were determined at 50°C and pH 5.0 (0.1 M Na-acetate buffer) using mutan (from *A. niger*) as a substrate. The substrate concentration was varied in the range of 0.1–5.0 g/L. The initial rates of the enzymatic reaction were determined by the releasing of RS (30) as described above; the values of *K*_m_ and *k*_cat_ were calculated by analyzing data obtained by the method of nonlinear regression using the OriginPro8 software. The experiments were conducted in triplicates.

### Enzyme thermostability studies

The solutions of purified MutA were incubated at pH 5.0 (0.1 M Na-acetate buffer) and 40, 50, 55 or 60°C in a thermostat Bio TDB 100 (Biosan, Latvia). After a certain incubation time, aliquots of the solution were taken and the residual activity against mutan (from *A. niger*) was determined under the standard conditions (see above). The initial activity of the enzyme sample was taken as 100%. The experiments were carried out in triplicates.

### Progress kinetics of mutan hydrolysis

Hydrolysis of mutan (from *A. niger,* 100 mg/mL) was carried out for 48 h at 50°C and pH 5.0 (0.1 M Na-acetate buffer) in 2 mL test tubes. Purified MutA was used for hydrolysis. The MutA dosage was 0.2, 1.0, and 10 units of activity per 1 g of mutan. After 3, 6, 24, and 48 h of the enzymatic reaction aliquots were taken from the reaction mixture, boiled at 100°C water bath for 5 min to terminate the reaction, and centrifuged to remove the denatured protein. The amount of RS released was determined by the modified Nelson-Somogyi method (31).

Products of mutan were determined by HPAEC-PAD using Agilent 1200 (Agilent Technologies, Santa Clara, CA) HPLC system equipped with binary pump, ALS, and ESA Coulochem III (Thermo Scientific, USA) electrochemical detector. CarboPac PA200 (Thermo Scientific, Waltman, MA) column with guard was used for the separation of the products of mutan hydrolysis, 0–450 mM sodium acetate gradient in 100 mM sodium hydroxide.

### Hydrolysis of cinnamoyl derivatives of oligosaccharides by purified MutA

Enzymatic hydrolysis of cinnamoyl derivative **6b** with a concentration in the reaction mixture of 100 µM was carried out under the action of a purified MutA (at a concentration of 0.08, 0.016, and 0.0016 mg/mL). Enzymatic hydrolysis of cinnamoyl derivative **1b–5b** and **7b** with concentration 200 µM was carried out at a concentration of MutA 0.016 mg/mL. A small amount of the substrate was dissolved in 100 µL of 20 mM Na-acetate buffer, pH 5.0; optical density of this solution was measured at a wavelength of 280 nm using a NanoDrop (Thermo Scientific, USA). The concentration of the sample was calculated from the value of 19,900 L*mol^−1^*cm^−1^ for the molar extinction coefficient of cinnamic acid and equalized by adding a calculated volume of 20 mM Na-acetate buffer. The analysis of the products of the enzymatic reaction was carried out using Agilent 1200 HPLC system (Agilent Inc., USA) equipped with refractive index and diode array detectors. All samples were dissolved in 20 mM Na acetate buffer and applied on a Cosmosil Sugar-D (Nacalai Tesque, Japan) column (250 mm × 3 mm × 5 µm; column temperature 35°C). An isocratic elution mode in acetonitrile:water with the addition of 20 mM Na-acetate buffer in the ratio 79:21 with flow rate 0.7 mL/min was used. Spectrophotometric detection was carried out at a wavelength of 276 nm with reference at 550 nm. Cycle analysis time was 17 min. Calibration was performed using *N*-cinnamoylated oligo-α-(1→3)-D-glucoside probes.

### Evaluation of degradation of bacterial biofilms

Biofilms were formed by clinical isolates of gram-positive (*Staphylococcus aureus* strain 15) and gram-negative (*Pseudomonas aeruginosa* strain 32*, Acinetobacter baumannii* strain 503*, Klebsiella pneumoniae* strain 1553*, E. coli* strain 717*, Salmonella typhimurium* strain C53) bacteria.

The formation of mature biofilms of the tested bacteria was carried out for 24 h at 37°C and pH 6.0 in the wells of 96-well plates (four repetitions of each variant) according to the modified form of technique by O’Toole (32). After removing planktonic cells from the wells, a solution of the mutanase enzyme preparation M22 (1 g/L) was added to the formed biofilms and kept for 1 h at 37°C and pH 6.0. Then, the mutanase solution was removed, and the biofilms were stained with a solution of gentian violet (crystal violet) dye, after that the biofilm was washed from the unbound dye. The dye bound to the components of biofilms was extracted with ethanol for 1 h, and then the color intensity of extracts was estimated using Varian 50 Cary UV-VIS spectrophotometer at a wavelength of 540 nm. Wells with native (primary) biofilms and wells with biofilms treated with water, saline solution, or solution of M22 enzyme preparation (1 g/L) produced by recipient *P. verruculosum* B-537 (*ΔniaD*) strain were used as controls. The results were evaluated statistically using the nonparametric Mann-Whitney test.

To study the effect of purified MutA on biofilms by means of fluorescence microscopy using Nikon H600L (Nikon, Japan), the biofilms of bacteria *S. aureus* 15 and *P. aeruginosa* 32 were formed on the surface of glass slides for 24 h at 37°C. Then, the slides were divided into two groups: (i) primary native biofilms were stained with solutions of gentian violet or alcian blue; (ii) native biofilms were treated with a MutA solution (with activity 1.5 U/mL) and then also stained.

## RESULTS

### Mutanase (MutA) sequence analysis

Gene *mutAW T. harzianum* consists of 1,999 bp. The coding part of the gene (1,902 bp) encodes a polypeptide of 634 amino acid residues (GenBank accession number HQ871941, which completely matches our sequence). The amino acid sequence for *T. harzianum* MutA and the gene sequence from which it was translated are shown in Fig. S3. This protein sequence has homology with other α-glucanases, but the highest sequence identity was observed in the case of murein transglycosylase from *T. harzianum* (Score 135, AN: KKP01663.1), glycoside hydrolase family 71 from *T. virens* Gv29-8 (Score 108, AN: XP 013954682.1), α-1,3-glucanases GH71 from *T. guizhouense* (Score 107, AN: OPB40799.1), and mutanase precursor from *T. harzianum* (Score 98, AN: ADZ45396.1).

In accordance with SignalP tool prediction (http://www.cbs.dtu.dk/services/SignalP), the first 24 amino acid residues of the MutA are assumed to be a signal peptide. The heterologous signal peptide was retained for the secretion of mutanase since the secretion of other *Trichoderma* proteins in the *P. verruculosum* strain proves this possibility (33).

The theoretical molecular mass and p*I* of the mature protein calculated from its amino acid composition without signal peptide by the Motif Scan tool (http://myhits.isb-sib.ch/cgi-bin/motif_scan) were 67,638 Da and 5.77, respectively.

### Heterologous expression of *mutA* gene

The *T. harzianum mutA* gene was cloned and heterologously expressed into *P. verruculosum* B1-537 (Δ*niaD*) host strain. Several clones of *P. verruculosum* with the highest activity of the fermentation broth against mutan were selected after the screening of the transformants after cultivation in shake flasks. The SDS-PAGE analysis of the fermentation broth (Fig. S4) showed that new protein band with a mass of ~70 kDa appeared in the case of a new *P. verruculosum* recombinant strains carrying the *mutA* gene, compared with the control sample based on the recipient *P. verruculosum* B1-537 (Δ*niaD*) host strain. The content of MutA was 40% of the total secreted protein (calculation was made on the basis of analysis of SDS-PAGE of the fermentation broth as well as protein chromatography data).

Selected after cultivation in shaking flasks best by the highest activity transformants (M18, M22, M25, and M37) were fermented in 3L fermenters. The dynamics of mutanase activity increase in the fermentation medium during fermentation are presented in Table S1. The mutanase activity at the end of fermentation of the best producer (M22) was 310 U/mL toward mutan from *A. niger*.

Specific activities of the mutanase enzyme preparations obtained after 3L fermentation (representing freeze-dried cultural filtrates) against different substrates are shown in Table 1, and the highest specific mutanase activity was observed for the clone M22 (10.7 U/mg of protein). The specific activity of the M22 enzyme preparation containing MutA is increased 150-folds compared to the activity of enzyme preparation obtained by *P. verruculosum* host strain under inducing conditions (0.07 U/mg, PV-control in Table 1). It should be noted that the specific activity of PV-control enzyme preparation in relation to MCC, reflecting the cellobiohydrolase activity, was 0.51 U/mg, while it decreased 0.22–0.30 U/mg in the series of enzyme preparations enriched the recombinant MutA. This phenomenon is related with the use of the *cbh1* gene promoter of the respective recipient strain for the heterologous expression of the recombinant MutA under study, resulting in a lower expression of CBH I by the new recombinant strains of *P. verruculosum* as we have previously demonstrated (34).

**TABLE 1:** Specific activities (U/mg of protein) of the mutanase enzyme preparations^*a*^ obtained in 3L fermenters toward different substrates

Clone number	Mutan (*A. niger*)	CMC	MCC	PNPG	Xylan
М18	3.0 ± 0.15	10.0 ± 0.50	0.30 ± 0.015	0.65 ± 0.032	12.0 ± 0.60
М22	10.7 ± 0.51	5.6 ± 0.25	0.30 ± 0.015	0.47 ± 0.021	6.2 ± 0.31
М25	6.6 ± 0.32	7.4 ± 0.35	0.22 ± 0.011	0.53 ± 0.05	3.4 ± 0.16
М37	4.6 ± 0.26	5.2 ± 0.24	0.24 ± 0.012	0.51 ± 0.023	9.1 ± 0.48
PV-control	0.07 ± 0.005	17.1 ± 0.95	0.51 ± 0.023	1.10 ± 0.051	18.1 ± 0.98

^*a*^ Data for the *T. harzianum* mutanase preparation are not presented because the mutanase content in it does not exceed 0.5%.

Specific activities of dry mutanase enzyme preparations after 3L fermentation toward CMC (endo-glucanase), PNPG (β-glucosidase), xylan (xylanase) were decreased compare with the same activities of PV-control preparation due to a decrease in the content of the enzymes responsible for the corresponding activities in the recombinant enzyme preparations.

### Purification of MutA

Enzyme preparation obtained after 3L fermentation of clone M22 was used for isolation and purification of MutA. Three stages of protein chromatography were used for the purification of the MutA. The enzyme was fractionated by using AEC on a Source 15Q column at pH 6.8 followed by HIC on a Source 15 Isopropyl column and finally polished using gel-filtration Superose 12 column (data not shown). SDS-PAGE data of purified MutA are shown in Fig. S5.

The MutA had an apparent molecular mass of 70 kDa and p*I* 5.8. The molecular weight of the mutanase observed on SDS-PAGE is greater than the theoretical weight which is explained by the presence of protein *N*-glycosylation (that has been proven by the use of *N*-Glycosidase F). The p*I* value determined by isoelectrofocusing was in good correlation with the theoretical value 5.77 (see above).

The amino acid sequence of MutA was confirmed by the MALDI-TOF MS of the tryptic hydrolyzate of the protein from the respective gel band. The resulting mass spectrum is shown in Fig. S6. Analysis of the mass spectrum revealed the presence of 11 peptides (with *m*/*z* 919.4, 1,359.5, 1,606.8, 1,660.8, 1,831.9, 2,355.2, 2,564.3, 3,150.5, 3,211.6, 3,548.8, 4,952.2 Da; in Fig. S3 shown in red and blue color) with masses corresponding to the theoretical ones calculated with the less than 100 ppm error. Thus, the obtained experimental mass spectrometry data confirmed the expression of the *mutAW T. harzianum* gene in the *P. verruculosum* B1-537 (Δ*niaD*) recipient strain.

### High specificity of the purified MutA against substrates containing α-(1→3)-glucosidic bonds

Specific activities of purified MutA toward different polymeric substrates are shown in Table 2. The enzyme displayed high activity toward α-(1→3)-glucosidic bonds in mutan (40 U/mg of protein). The MutA also displayed trace activity toward CMC (contains β-(1→4)-glucosidic bonds), barley β-glucan (contains β-(1→3/4)-glucosidic bonds), and pachyman (contains β-(1→3)-glucosidic bonds)—0.01–0.02 U/mg of protein probably due to the minor cellulases impurities. Activities toward laminarin (contains β-(1→3)-glucosidic bonds), pustulan (contains β-(1→6)-glucosidic bonds), xylan (contains β-(1→4)-xylosidic bonds), dextran (contains α-(1→6)-glucosidic bonds), and starch (contains α-(1→4)-glucosidic bonds) was equal to zero.

**TABLE 2:** Specific activities of purified MutA toward different substrates

Substrate	Bond	Activity, U/mg
Mutan (*A. niger*)	α-(1→3)	40 ± 2.0
β-Glucan	β-(1→3/4)	0.022 ± 0.001
CMC	β-(1→4)	0.02 ± 0.001
Pachyman	β-(1→3)	0.012 ± 0.001
Laminarin	β-(1→3)	0
Xylan (birch wood)	β-(1→4)	0
Dextran	α-(1→6)	0
Starch	α-(1→4)	0

*K*_m_ and *k*_cat_ for MutA were determined in the hydrolysis of mutan. *K*_m_ and *k*_cat_ were of 1.0 g/L and 30 s^−1^, respectively (pH 5.0, 50°C).

### Effect of pH and temperature on the activity and stability of purified MutA

The maximum activity of MutA toward mutan was observed at pH 5.0. The MutA displayed bell-shaped form of the enzyme activity dependence on pH, and more than 80% of the activity was retained at pH 4.4–5.6. The temperature optimum for MutA was 50°C observed at pH 5.0, and more than 80% of the activity was retained at 47–62°C. The observed pH and temperature optima for the enzyme was like those reported for other fungal mutanase (35).

Thermostability studies showed that the MutA was 100% stable at 40°C and pH 5.0: no decrease in activity was observed during at least 3 h of incubation; while at 50°C, the enzyme retained 83% of the initial activity after 3 h of incubation. At 55 and 60°C, the half-life time of MutA was 10 min and 5 min, respectively.

### Progress kinetics of mutan hydrolysis shows endo-processive type of mutan destruction by MutA

**Fig 2:**
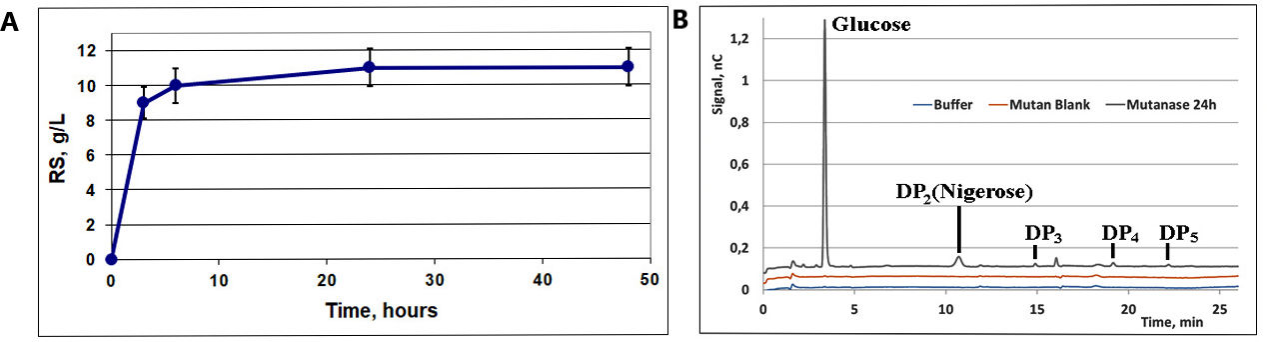
Mutan hydrolysis by the purified MutA. (**A)** Progress kinetic curves (RS release) for 10 units of MutA per 1 mL of the reaction mixture.** (B)** HPAEC-PAD evaluation of hydrolysis products after 24 h of reaction (10 U/mL of mutanase activity).

Progress kinetics of mutan (100 g/L) hydrolysis by the purified MutA at different enzyme activity loading in the reaction mixture (0.2–10 U/mL) was studied. The maximum concentration of RS in the reaction mixture for MutA dosage 10 U/mL after 48 h was 11 g/L, i.e., the degree of mutan conversion did not exceed 10% (Fig. 2A).

Glucose was the main product (72%–79% depending on enzyme dosage) of mutan hydrolysis, the content of α-1,3-disaccharide (nigerose) was in the range 3.8%–5.7%, and the content of oligosaccharides with a higher degree of polymerization was low (see HPAEC-PAD data, Fig. 2B).

### Synthetic oligosaccharide probes bearing UV-detectable cinnamoyl residue hydrolysis data confirms that MutA is an endo-processive type of enzyme

The substrate specificity of GH71 mutanase was studied with a series of synthetic oligo-α-(1→3)-D-glucoside probes bearing UV-detectable cinnamoyl residue. Along with these probes consisting of one to seven α-D-glucosyl residues (**1–6**), the labeled penta-α-(1→3)-D-glucoside (**7**) bearing *N*-acetyl-β-D-glucosaminyl residue at non-reducing end were used. The labeled synthetic oligosaccharide probes were shown (36–39) to be convenient instruments in the investigation of carbohydrate specificity of polysaccharide hydrolases and glycosyl transferases. Modern methods of stereoselective α-D-glucosylation (40–42) permit the efficient synthesis of oligo-α-(1→3)-D-glucosides. That is why the study of substrate specificity of GH71 mutanase described in present work was performed with a use of a series of synthetic oligo-α-(1→3)-D-glucoside probes (**1b–7b**) bearing UV-detectable *N*-*trans*-cinnamoyl residue in the aglycon (see Fig. 1). Along with these probes consisting from one to seven α-D-glucosyl residues, only the labeled penta-α-(1→3)-D-glucoside derivative (**7b**) bearing *N*-acetyl-β-D-glucosaminyl residue at non-reducing end was also used.

**Fig 3:**
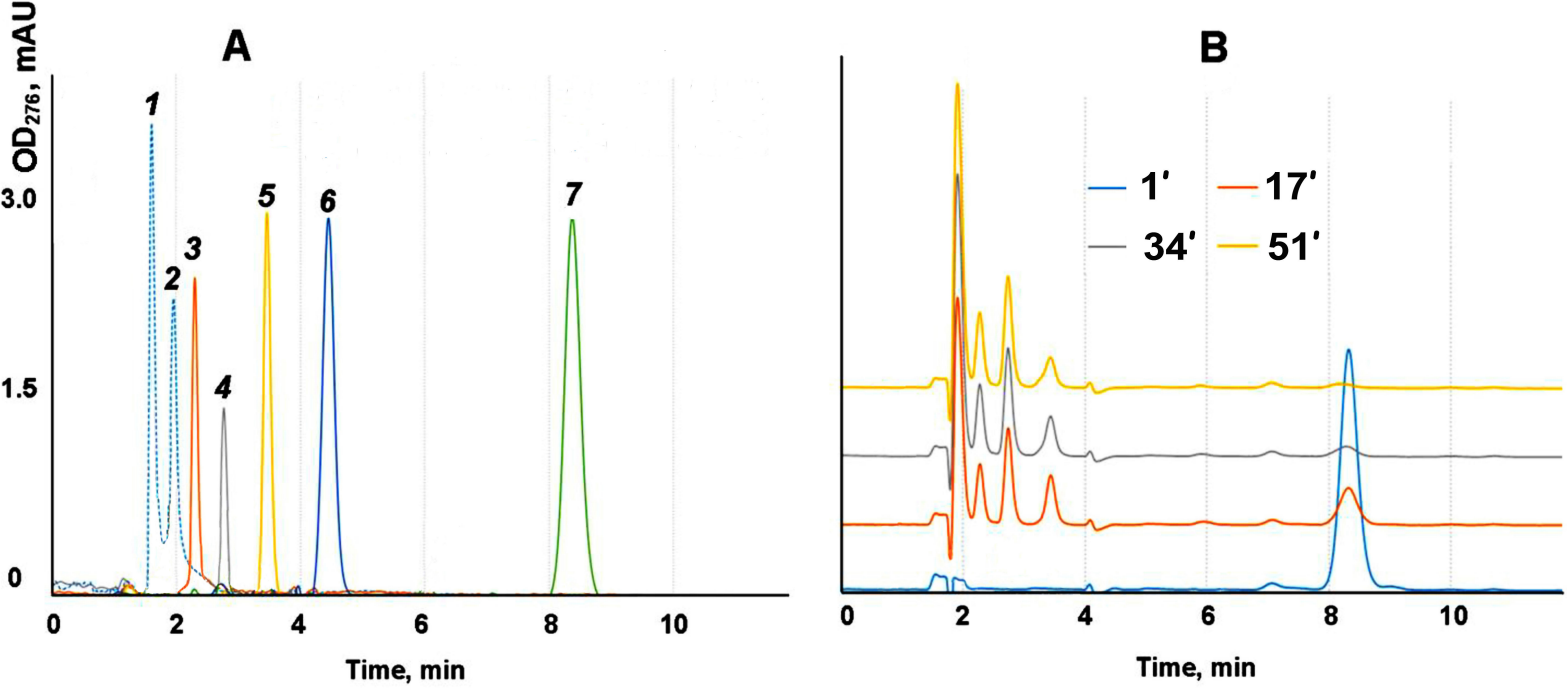
(A) Chromatograms of cinnamoylated substrates **1b–7b** and (**B)** chromatograms of oligosaccharide **6b** and the products of its hydrolysis by MutA at a concentration of 0.016 mg/mL (CH_3_CN:H_2_O 79:21). *1*—Cinnamic acid (5.03 OE); *2*–**1b** (5.03 OE); *3*–**2b** (3.52 OE); *4*–**3b** (2.79 OE); *5*–**4b** (4.88 OE); *6*–**5b** (2.38 OE); *7*–**6b** (3.52 OE).

**Fig 4:**
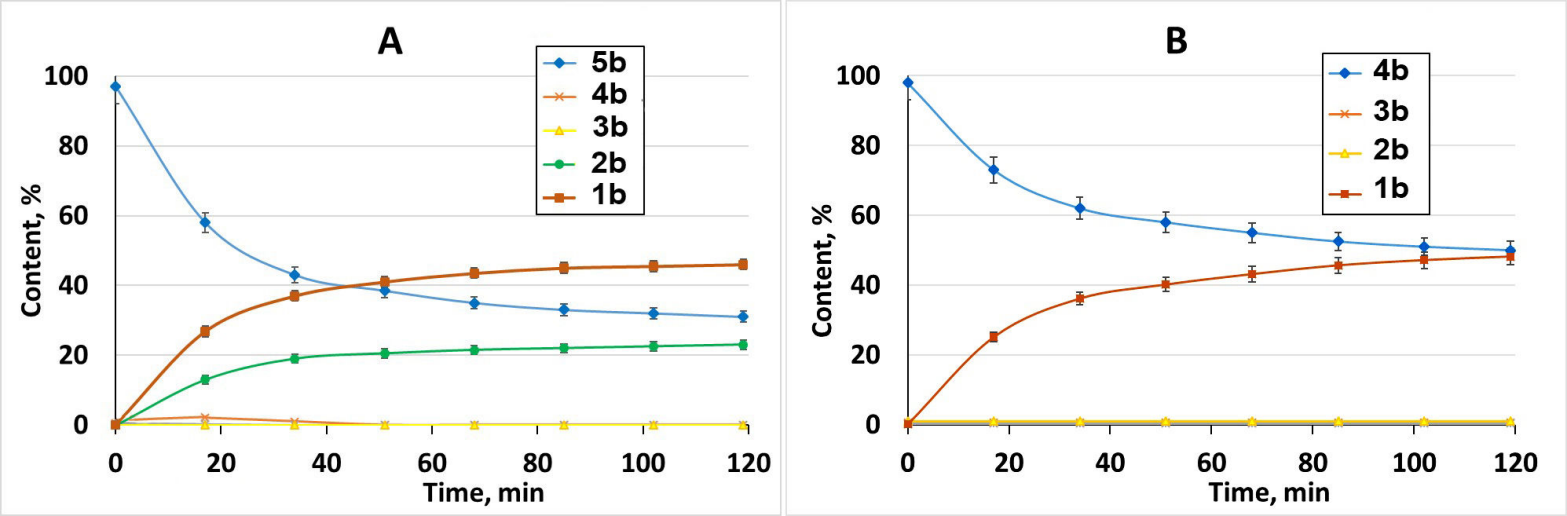
Kinetics of 200 µM **5b** (**A**) and 200 µM **4b** (**B**) hydrolysis by MutA at a concentration of 0.016 mg/mL.

The mechanism and kinetics of the enzymatic hydrolysis of synthetic substrate cinnamoyl derivative of oligosaccharides by purified MutA were studied by means of HPLC. Retention times calibration was performed using cinnamic acid and cinnamoyl-derived oligosaccharides with the degree of polymerization from 1 to 7 (Fig. 3A). Sampling and analysis of the reaction mixture were carried out every 17 min. After 17 and 34 min, nothing remains of the original **6b** substrate at MutA concentration of 0.08 mg/mL. Fig. 3B shows the chromatographic profiles of **6b** hydrolysis by purified MutA at a concentration of 0.016 mg/mL. A gradual decrease of the **6b** peak and an increase of peaks of low-molecular-weight reaction products first of all of mono- and disaccharide derivatives **1b** and **2b**. A decrease in concentration of MutA by an order of magnitude (to 0.0016 mg/mL) led to a slower decrease of **6b** peak, with a simultaneous increase of **1b** and **2b**. The concentration of the enzyme of 0.016 mg/mL was chosen to observe the hydrolysis kinetics in a reasonable time (up to 2 h) for cinnamoyl derivatives **1b–5b** to study the preference of the MutA in the substrate polymerization degree. Kinetics of **5b** and **4b** substrates hydrolysis by MutA (at a concentration of 0.016 mg/mL) is presented in Fig. 4A and B. **5b** was hydrolyzed to give **1b** as the main product, **2b**—twice less, **4b** and **3b** remain on the same level during the reaction (Fig. 4A). The rate of **4b** hydrolysis (Fig. 4B) was lower compared to **5b**. **1b** was detected as the main product of the reaction. A very limited (less than 2% after 2 h) decrease of **3b** was observed at the same reaction conditions to give **1b** as a product, while practically no hydrolysis was observed in the case of substrates **1b** and **2b** (data not shown). The main product of **7b** treatment by MutA (at a concentration of 0.016 mg/mL) is a monosaccharide **1b**, while disaccharide **2b** is formed about 4.2 times less (Fig. S7 ).

### Effective destruction of biofilms by mutanase enzyme preparation and purified MutA

The activity of the MutA enzyme preparation M22 was tested against biofilms of *S. aureus* (gram-positive) and *P. aeruginosa, A. baumannii, K. pneumoniae, E. coli, S. typhimurium* (gram-negative) bacteria using crystal violet dye staining technique (this dye mainly stains bacteria and extracellular DNA in biofilms organized into a matrix). Biofilms were treated for 1 h at 37°C and pH 6.0.

**Fig 5:**
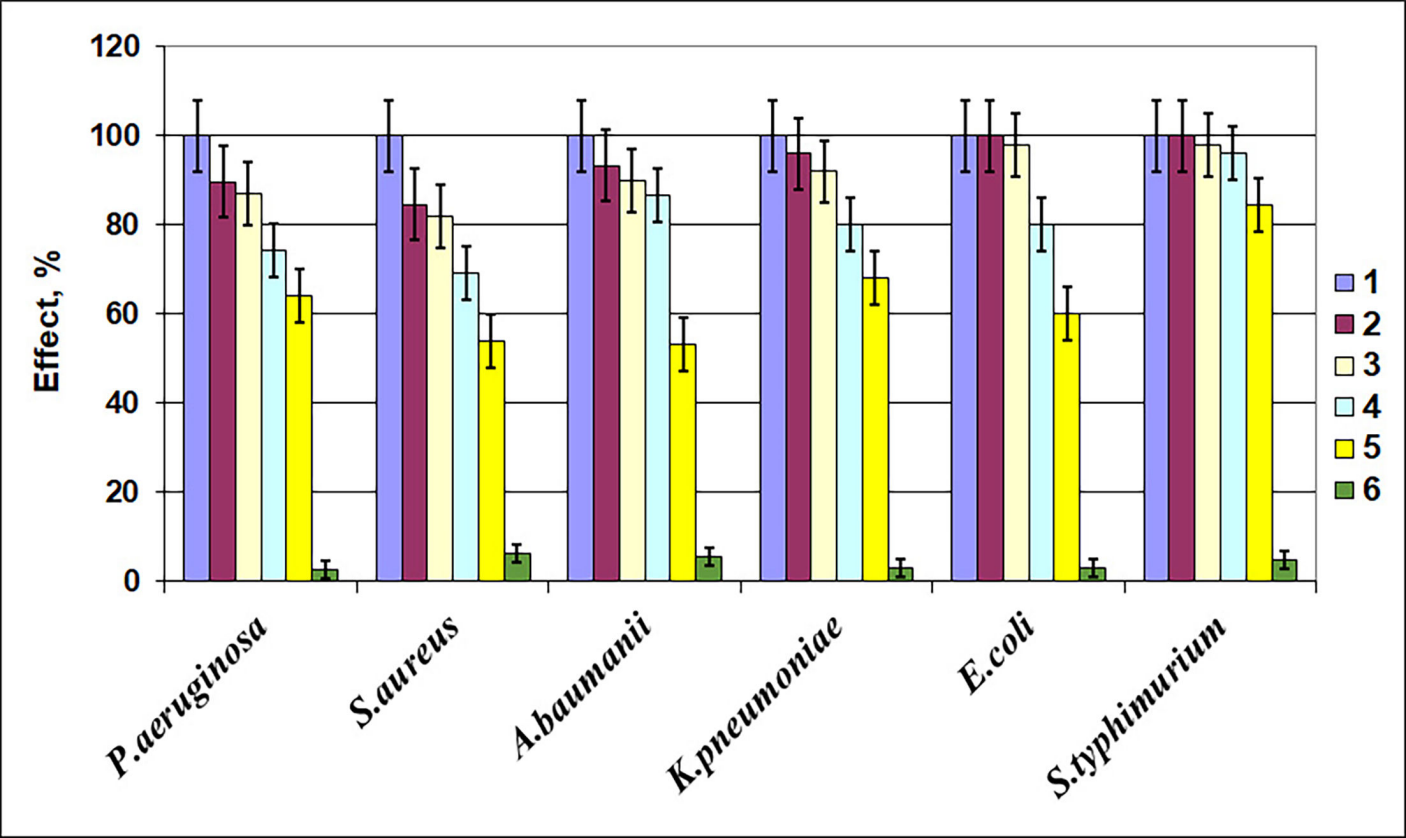
The effect of mutanase enzyme preparation M22 on biofilms of various types of microorganisms (staining with gentian violet in small wells of a 96-well plate): *1*—primary biofilm; *2*—primary biofilm and distilled water; *3*—primary biofilm and inactivated mutanase enzyme preparation M22; *4*—primary biofilm and saline solution; *5*—primary biofilm and *P. verruculosum* B1-537 cellulase enzyme preparation produced by recipient strain under inducing conditions (control); *6*—primary biofilm and mutanase enzyme preparation M22.

The water, saline solution, or solution of inactivated mutanase enzyme preparation M22 (1 g/L) partly contributed to a decrease in the intensity of the extracted color (see Fig. 5, columns 2–4). Solution of dry enzyme preparation (1 g/L) produced by recipient *P. verruculosum* B-537 strain under induction by MCC conditions also lead to some decrease in the intensity of the extracted color (Fig. 5, column 5, activity of this preparation corresponds to PV-control in Table 1). Solution of mutanase-enriched enzyme preparation M22 (1 g/L) obtained under induction conditions when the strong *cbh1* promoter utilized for mutanase expression caused a significant increase in mutanase activity (the activities of M22 preparation given in Table 1) leads to an obvious and very significant decrease in the staining intensity of the biofilms for both gram-negative and gram-positive bacteria extracts (Fig. 5, column 6). What was discovered in these experiments indicates a significant violation of the integrity of the biofilms structure because of the destruction of the polysaccharide backbone of the biofilms with subsequent leaching of extracellular DNA and bacteria. This indicates a significant violation of the integrity of the biofilms structure because of the destruction of the polysaccharide backbone of the biofilms with subsequent leaching of extracellular DNA and bacteria.

**Fig 6:**
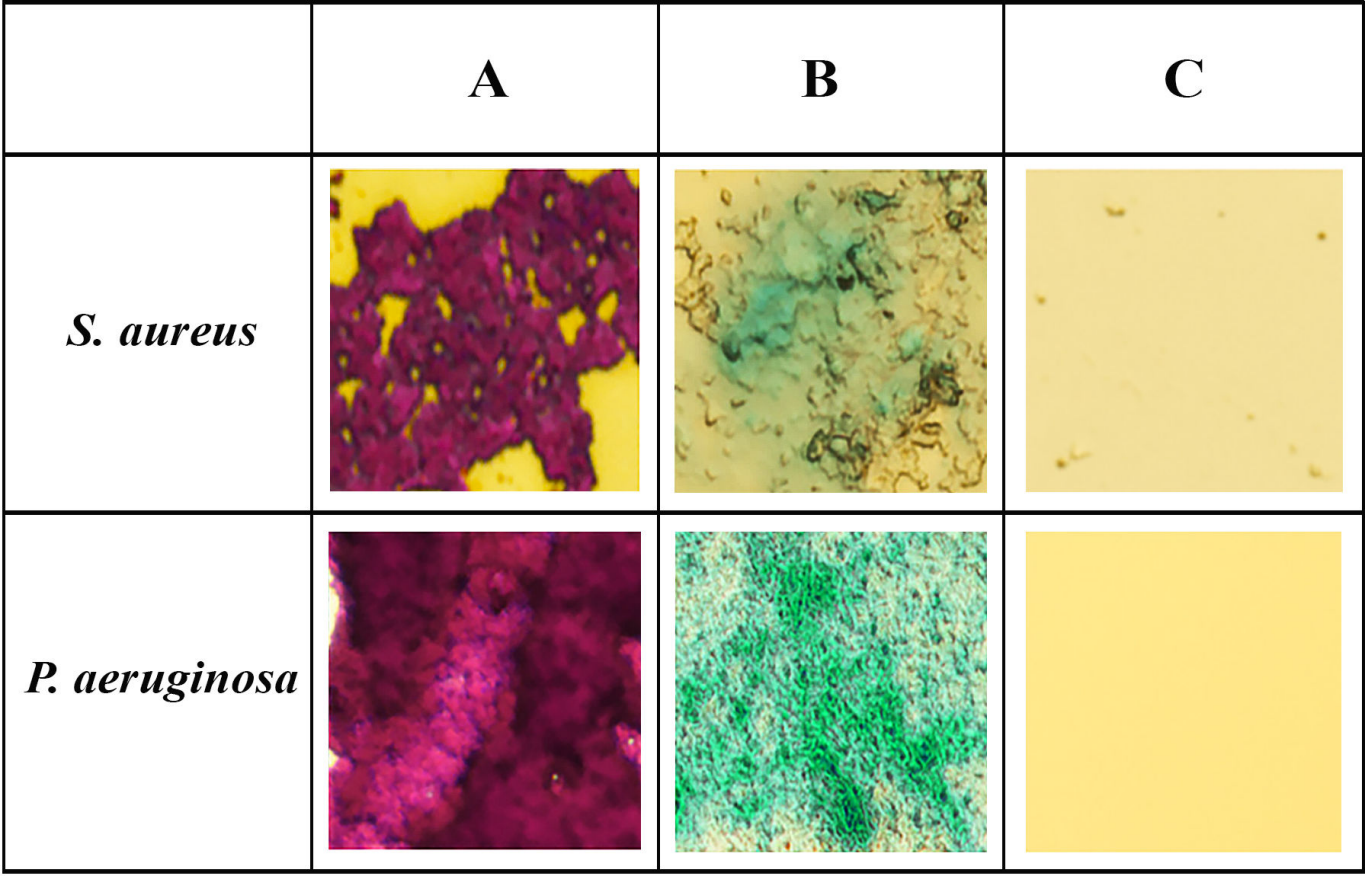
Visualization of the effect of purified MutA on the primary biofilms produced by *S. aureus* and *P. aeruginosa* on the surface of glass slides and stained with gentian violet (**A**) and alcian blue (**B**) primary biofilms and purified MutA (**C**).

Furthermore, the high efficiency of purified MutA action on polysaccharide biofilms was also confirmed in experiments with biofilms of *S. aureus* 15 and *P. aeruginosa* 32 formed on the surface of glass slides dye staining techniques and fluorescence microscopy. Primary (native) biofilms and biofilms treated with a MutA solution (1.5 U/mL) were stained with two dyes—gentian violet and alcian blue (this dye predominantly stains the polysaccharide components of biofilms). The results of the experiment are shown in Fig. 6. When stained with gentian violet, the *P. aeruginosa* biofilms look more intensely colored than the *S. aureus* biofilms. Apparently, the disruption of the polysaccharide backbone of the biofilms because of the action of MutA promotes the release and subsequent elution of both bacteria and extracellular DNA bound in the composition of the biofilms with the bacterial surface and positively charged components of the biofilms (possibly with Ca^2+^ cations). Similar results were obtained for staining with alcian blue: MutA solution destroyed the polysaccharide matrix of both gram-positive (*S. aureus*) and gram-negative bacteria (*P. aeruginosa*).

## DISCUSSION

### Sequence analysis of MutA

Analysis of the MutA sequence using software package on the ExPASy Proteomic Server (http://ca.expasy.org/tools/dna.html) and comparison of nucleotide and amino acid sequences (Fig. S3) with those for other enzymes belonging to the same protein family showed the presence of two introns (53 and 44 bp) in the *mutAW* gene. BLAST analysis showed that the enzyme displays similarity to GH71 α-1,3-glucanases. So, it was classified as glycoside hydrolase family 71 (GH71, mutanase, MutA, (http://www.cazy.org/GH71.html).

### Transformation of *P. verruculosum* host strain

To transform the *P. verruculosum* host strain, the pSTA10 plasmid contained a native nitrate reductase (*niaD*) gene. The *P. verruculosum* host strain is an auxotroph, i.e., has a defect in the *niaD* gene. This disrupts the metabolic pathway for the assimilation of nitrate nitrogen and makes the growth of host strain on media supplemented with sodium nitrate impossible. When the pSTA10 plasmid is transformed with the target pCBHI-Mut plasmid, the recombinant strains return to prototrophy, which makes it possible to select resulting transformants on media with sodium nitrate (NaNO_3_, 10 mM).

### Heterologous expression of MutA

Both homologous and heterologous expressions of mutanases were reported in literature. In order to increase the level of homologous expression of the enzyme, the mutan is normally used (43). In our work to produce the MutA from *T. harzianum,* we applied the *P. verruculosum* fungus expression system, which is based on the use of a strong inducible *cbhI* promoter combined with *mutA* gene. In this case, microcrystalline cellulose (MCC) played the role of the inducer of the biosynthesis of heterologous mutanase.

Another peculiarity of heterologous expression is post-translational modification of the protein. For example, the actual molecular masses of recombinant α-1,3-glucanases from *T. harzianum* and *P. purpurogenum* (86 and 90 kDa, respectively) were greater than the theoretical ones (about 63 kDa) (44, 45). The same picture was found in our case for the MutA: the molecular weight of the mutanase observed on SDS-PAGE was greater than the theoretical weight which is explained by the presence of protein *N*-glycosylation.

According to reference 45, mutanase from *T. harzianum* (MutAp) consists of a N-terminal signal sequence, catalytic domain, Thr-Pro-Ser rich-linker, and carbohydrate-binding module family 24 (CBM 24). The C-terminal CBM24 domain shows insoluble α-1,3-glucan binding activity, which suggests that this domain is useful for capturing extracellular α-1,3-glucan in nature and *T. harzianum* efficiently obtains nutrition from α-1,3-glucan. An α-1,3-glucanase with a similar multidomain structure has been found in *P. purpurogenum*.

### Reaction mechanism of MutA

Based on the gene sequence analysis MutA *T. harzianum* was classified as glycoside hydrolase family 71. According to NCBI data, GH71 α-1,3-glucanases appear to have an endo-hydrolytic mode of enzymatic activity (https://www.ncbi.nlm.nih.gov/Structure/cdd/cd11577). The theoretical prediction was confirmed by the analysis of reaction products from the substrates, such as mutan and reducing end-labeled oligosaccharide (Fig. 1, **1b-7b**). MutA is an endo-processive type of enzyme. For long-chain substrates, MutA cleaved random internal linkages. Subsequently, the enzyme hydrolyzed and released glucose from the reducing end and slides into the non-reducing end. The hydrolysis continues until tri-saccharide remains. The results obtained correlate quite well with the literature data. For example, by Grun et al., it was demonstrated (46) that fungal GH71 mutanases (MutAp of *T. harzianum*) is endo-hydrolytic enzyme and progressively hydrolyzes α-(1→3)-d-glucan releasing glucose from the reducing and recognizes tetra-saccharide as a minimal substrate (for long-chain substrates, they cleave random internal linkages after binding to the substrate).

It is worth noting especially the important role of the synthetic substrate cinnamoyl derivative of oligosaccharides. The *N*-cinnamoyl group offers high sensitivity in detecting conjugates 1b–7b. However, when handling related derivatives, careful measures are necessary owing to the observed sluggish degradation. While the mechanism and products resulting from this degradation have not been studied by us, they could be caused by photoisomerization or oxidation of the tag in aqueous solutions.

### Effective destruction of bacteria biofilms by MutA

Since mutanases have the ability to hydrolyze α-1,3-glucosidic bonds in bacterial mutans, they could be applied as active ingredients in the mixtures for fighting bacterial films (4–6). As it was shown in the experiments with biofilms of various types of microorganisms (Fig. 5 and 6), MutA itself as well as crude enzyme preparations enriched with the enzyme were able to effectively destroy the polysaccharide backbone of the biofilms of clinical isolates of gram-positive and gram-negative bacteria.

Thus, we can suggest that the enzyme (MutA) wash could be useful to clean up medical devices and other surfaces since the bacterial infection of medical equipment can have the catastrophic complications and the consequences and at present careful attention to surgical technique and the use of perioperative antibiotics are the mainstay approaches.

### Conclusions

A novel recombinant MutA was purified in a homogeneous form. The enzyme exhibited a high activity against mutan and negligible or zero activity toward other types of glucans including α-(1→4)-, β-(1→3)-, β-(1→4), and β-(1→6)-glucans. The main reaction product of a long-term mutan hydrolysis by MutA was glucose. MutA is an endo-processive type of enzyme, which hydrolyzes the internal glucosidic bonds and releases glucose from the reducing end sliding into the non-reducing end. MutA recognizes tetrasaccharide as a minimal substrate and hydrolyzes it to trisaccharide and glucose. The effectiveness of the use of MutA enzyme preparation M22 and purified MutA for the destruction of clinical isolates of gram-positive and gram-negative bacteria was demonstrated.

## Data Availability

The DNA sequence encoding the mutanase (accession number HQ871941) of the fungus *Trichoderma harzianum* was deposited in the NCBI database (https://www.ncbi.nlm.nih.gov/Structure/cdd/cd11577).

## SUPPLEMENTAL MATERIAL

Supplemental material aem.00226-24-s0001.docx Figures S1 to S7; Table S1.

ASM does not own the copyrights to Supplemental Material that may be linked to, or accessed through, an article. The authors have granted ASM a non-exclusive, world-wide license to publish the Supplemental Material files. Please contact the corresponding author directly for reuse.
