# Supplementary material for: Physico-chemical properties and substrate specificity of α-(1→3)-d-glucan degrading recombinant mutanase from Trichoderma harzianum expressed in Penicillium verruculosum
Source: Appl Environ Microbiol. 2025 Jan 23;91(2):e00226-24. doi: 10.1128/aem.00226-24 (PMC11837517; doi:10.1128/aem.00226-24)
Supplement: Supplemental material — Figures S1 to S7; Table S1. [file aem.00226-24-s0001.docx]

**Physico-chemical properties and substrate specificity of α-(1→3)-D-glucan degrading recombinant mutanase from *Trichoderma harzianum* expressed in *Penicillium verruculosum***

O.A. Sinitsyna^1*^, P.V. Volkov^1^, I.N. Zorov^1,2^, A.M. Rozhkova^2^, O.V., Emshanov^3^, Yu.M. Romanova^4^, B.S. Komarova^5^, N. S. Novikova^5^, N.E. Nifantiev^5^, A.P. Sinitsyn^1,2^

^1^M. V. Lomonosov Moscow State University, Department of Chemistry

^2^Federal Research Centre «Fundamentals of Biotechnology» of the Russian Academy of Sciences

^3^LLC BFR Laboratories

^4^ The National Research Center for Epidemiology and Microbiology named after Honorary Academician N.F. Gamaleya of the Ministry of Health of the Russian Federation

^5^ Laboratory of Glycoconjugate Chemistry, N.D. Zelinsky Institute of Organic Chemistry Russian Academy of Sciences

**Supplementary Information**

**Contents:**

**S1.** Genetic map of expression plasmid with *mutA* gene under strong and inducible CBHI promoter.

**S2**. NMR Spectra, compounds **1b**-**7b**

**S3.** Nucleotide sequence for *T. harzianum* MutA (A), translated amino acid sequence for *T. harzianum* MutA (B). Matching tryptic peptides identified by MALDI-TOF MS are shown in red and blue color.

**S4.** The SDS-PAGE of the crude enzyme preparations (culture filtrates) produced by recombinant strains M18 (*1*), M22 (*2*), M25 (*3*) and M37 (*4*) of *P. verruculosum*, carrying the *mutA* gene, and the control sample (*5*) produced by *P. verruculosum* B537 (ΔniaD) host strain.

**S5**. SDS-PAGE of purified MutA.

**S6.** MALDI-TOF mass spectrum of the target MutA recorded on an UltrafleXtreme II instrument. Designations: a.u., arbitrary units.

**S7.** HPLC profile and kinetics of **7b** hydrolysis. Designations: OD_276_, optical density at 276 nm.

**Table S1**. Table Activity (U/ml) in the fermentation medium during the growing of the recombinant strains of *P. verruculosum*, carrying the *mutA* gene, and of the host strain *P. verruculosum* B537 (ΔniaD)


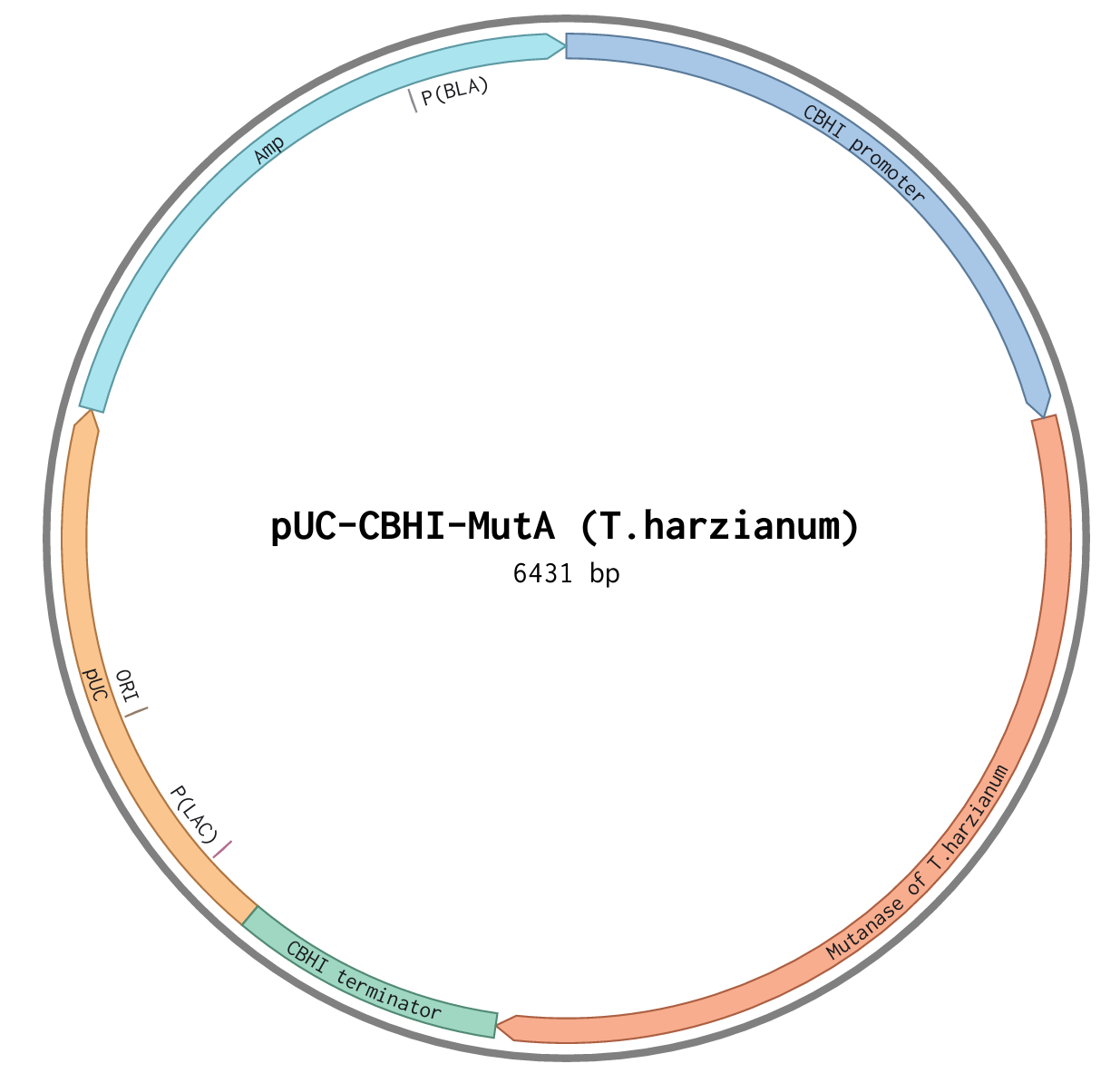


**FIG S1.** Genetic map of expression plasmid with *mutA* gene under strong and inducible CBHI promoter.

**S2**. NMR Spectra, compounds **1b**-**7b**

3-N-*trans*-Cinnamidopropyl α-d-glucopyranoside (**1b**). *R*_f_ 0.18 (CHCl_3_–MeOH, 5.5:1); [α]_D_^24^ = +62.9 (D_2_O, c 0.2). ^1^H NMR (600 3.59–3.53 (OC*H*_2_CH_2_CH_2_NHCinn(B), H-2), 3.43 (m, 2H, OCH_2_CH_2_C*H*_2_NHCinn), 3.39 (t, 1H, *J*_3,4_ = *J*_3,2_ 9.4 Hz, H-4), 1.92 (m, 2H, OCH_2_C*H*_2_CH_2_NHCinn). ^13^C NMR (150.9 MHz, CDCl_3_): δ_C_ 216.1 (CO), 141.7 (*C*Hβ (Cinn)), 130.9 (*m*-Ph), 129.7 (*p*-Ph), 128.6 (*o*-Ph), 120.9 (*C*Hα (Cinn)), 98.8 (C-1), 73.8 (C-3), 72.5 (C-5), 72.0 (C-2), 70.2 (C-4), 66.1 (O*C*H_2_CH_2_CH_2_NHCinn), 61.2 (C-6), 37.4 (OCH_2_CH_2_*C*H_2_NHCinn), 28.8 (OCH_2_*C*H_2_CH_2_NHCinn). C_18_H_25_NO_7_ 368.1704; found 368.1695.


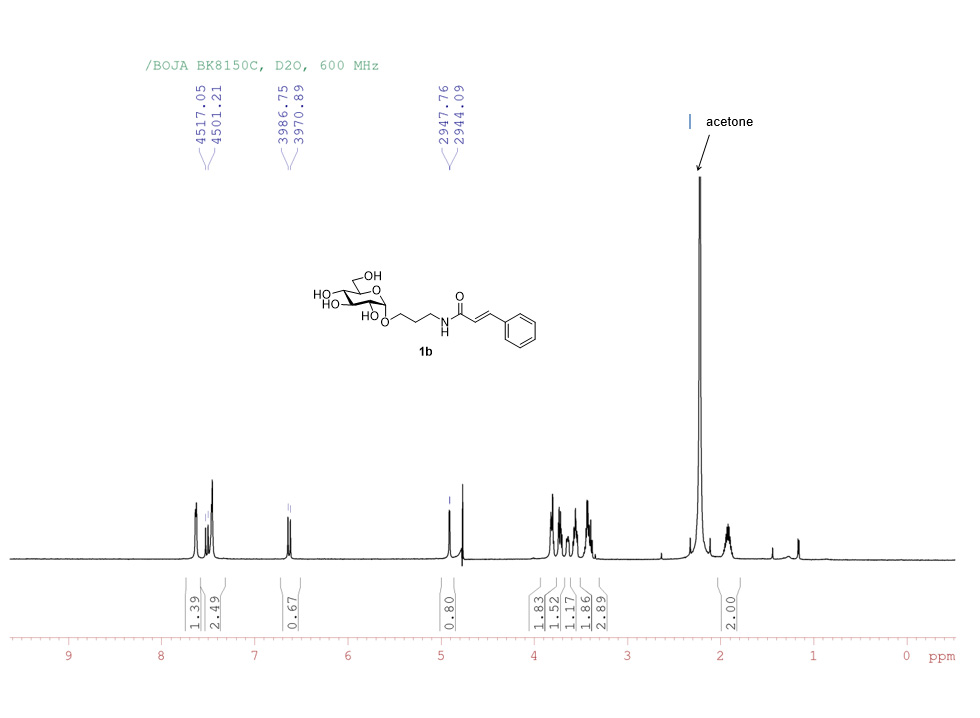


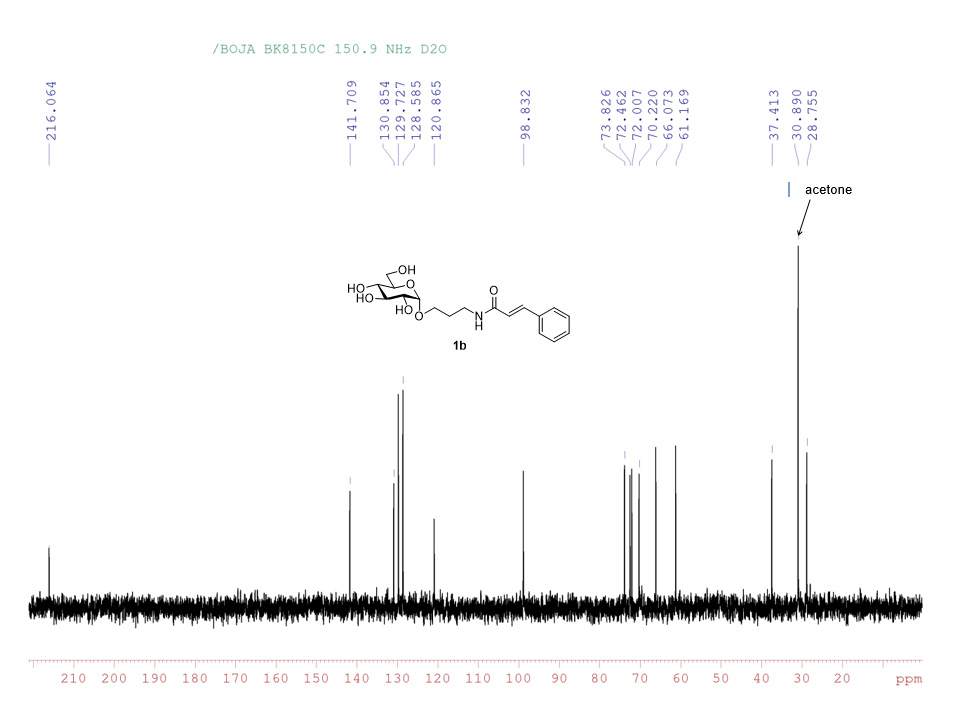


**3-N-*trans*-Cinnamidopropyl α-d-glucopyranosyl-(1→3)-α-d-glucopyranoside** **(2b)** *R*_f_ 0.49 (BuOH–EtOH–H_2_O–NH_3_aq., 5:5:4:1); [α]_D_^24^ = +117.8 (D_2_O, c 0.21). ^1^H NMR (600 MHz, D_2_O): δ 7.62–7.57 (m, 2H, *o*-Ph), 7.49 (d, *J*_β,α_ 15.8 Hz, CHβ (Cinn)), 7.46–7.40 (m, 3H, *m*-Ph, *p*-Ph), 6.60 (d, *J*_α,β_ 15.9 Hz, CHα (Cinn)), 5.21 (d, 1H, *J*_1,2_ 4.0 Hz, H-1^B^), 4.88 (d, 1H, *J*_1,2_ 3.8 Hz, H-1^A^), 3.94 (m, 1H, H-5^B^), 3.83–3.75 (m, 4H, H-3^A^, H-6A^A,B^, OC*H*_2_CH_2_CH_2_NHCinn(A)), 3.74–3.66 (m, 3H, H-3^B^, H-6B^A,B^), 3.64–3.52 (m, 4H, H-2^A^, H-4^A^, H-5^A^, OC*H*_2_CH_2_CH_2_NHCinn(B)), 3.46–3.33 (OCH_2_CH_2_C*H*_2_NHCin, H-2^B^, H-4^B^), 1.90 (m. 2H, OCH_2_C*H*_2_CH_2_NHCinn). ^13^C NMR (150.9 MHz, CDCl_3_): δ_C_ 142.3 (*C*Hβ (Cinn)), 131.4 (*m*-Ph), 130.2 (*p*-Ph), 129.2 (*o*-Ph), 120.9 (*C*Hα (Cinn)), 100.4 (C-1^B^), 99.5 (C-1^A^), 81.6 (C-3^A^), 74.0 (C-3^B^), 73.2 (C-5^B^), 72.8 (C-2^B^), 72.7 (C-5^A^), 71.1 (C-2^A^, C-4^A^), 70.7 (C-4^B^), 67.0 (O*C*H_2_CH_2_CH_2_NHCinn), 61.7 (C-6^B^), 61.5 (C-6^A^), 38.4 (OCH_2_CH_2_*C*H_2_NHCinn), 29.2 (OCH_2_*C*H_2_CH_2_NHCinn). HRMS ESI m/z calculated for [M+Na]^+^ C_24_H_35_NO_12_ 552.2052; found 552.2051.


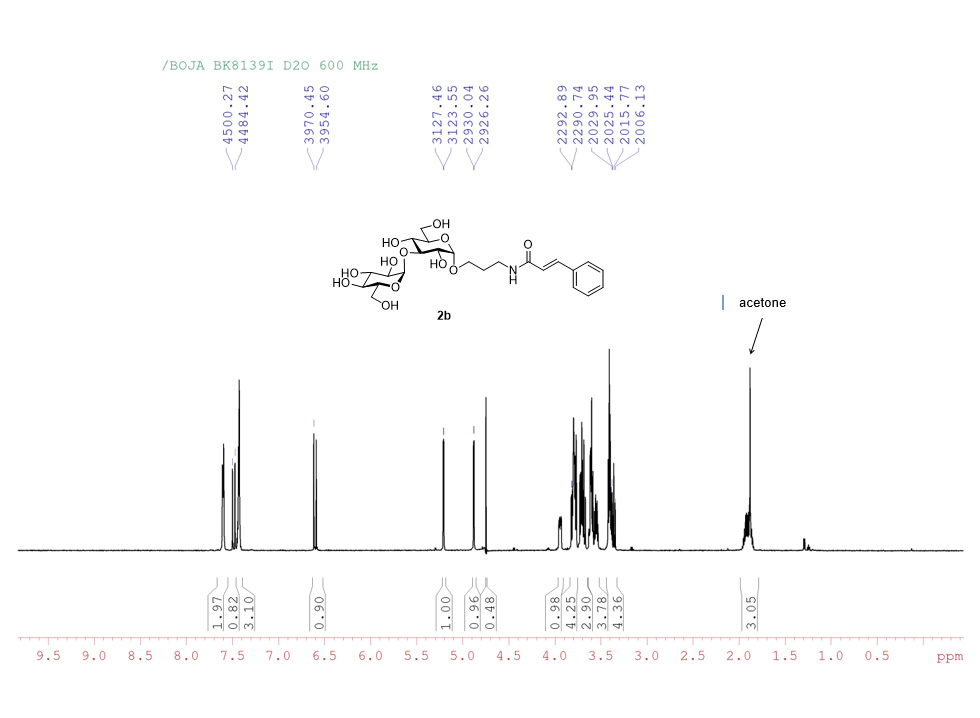

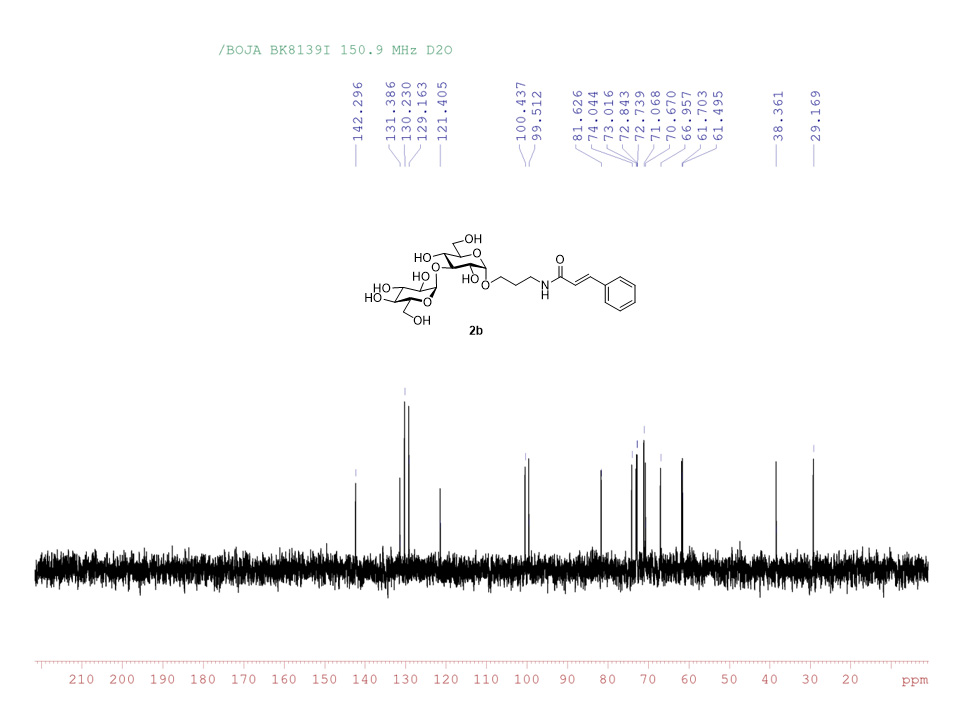


**3-N-*trans*-Cinnamidopropyl α-d-glucopyranosyl-(1→3)-α-d-glucopyranosyl-(1→3)-α-d-glucopyranoside (3b)** *R*_f_ 0.74 (BuOH–EtOH–H_2_O–NH_3_aq., 5:5:4:0.2); [α]_D_^24^ = +154.9 (D_2_O, c 0.33).  ^1^H NMR (600 MHz, D_2_O): δ 7.66–7.63 (m, 2H, *o*-Ph), 7.53 (d, *J*_β,α_ 15.9 Hz, CHβ (Cinn)), 7.50–7.46 (m, 3H, *m*-Ph, *p*-Ph), 6.65 (d, *J*_α,β_ 15.9 Hz, CHα (Cinn)), 5.35 (d, 1H, *J*_1,2_ 3.7 Hz, H-1^C^), 5.27 (d, 1H, *J*_1,2_ 3.9 Hz, H-1^B^), 4.93 (d, 1H, *J*_1,2_ 3.8 Hz, H-1^A^), 4.03–3.98 (m, 2H, H-5^C,B^), 3.87 (t, 1H, *J*_3,2_ = *J*_3,4_ 9.1Hz, H-3^B^), 3.86–3.80 (m, 5H, H-3^A^, H-6A^A-C^, OC*H*_2_CH_2_CH_2_NHCinn(A)), 3.79–3.72 (m, 4H, H-3^C^, H-6B^A-C^), 3.68–3.54 (m, 7H, H-2^A^, H-5^A^, H-4^A,B^, OC*H*_2_CH_2_CH_2_NHCinn(B), H-2^C,B^), 3.47–3.41 (m, 3H, H-4^C^, OCH_2_CH_2_C*H*_2_NHCinn), 1.94 (m, 2H, OCH_2_C*H*_2_CH_2_NHCinn). ^13^C NMR (150.9 MHz, CDCl_3_): δ_C_ 142.3 (*C*Hβ (Cinn)), 131.4 (*m*-Ph), 130.2 (*p*-Ph), 129.2 (*o*-Ph), 121.5 (*C*Hα (Cinn)), 100.6 (C-1^B^), 100.4 (C-1^C^), 99.6 (C-1^A^), 81.8 (C-3^A^), 81.2 (C-3^B^), 74.1 (C-3^C^), 73.0 (C-5^B,C^), 72.8 (C-2^C^), 72.8 (C-5^A^), 71.6 (C-2^B^), 71.2, 71.1 (C-2^A^, C-4^A,B^), 70.7 (C-4^C^), 67.0 (O*C*H_2_CH_2_CH_2_NHCinn), 61.7, 61.6 (C-6^A-C^). 38.4 (OCH_2_CH_2_*C*H_2_NHCinn), 29.2 (OCH_2_*C*H_2_CH_2_NHCinn). HRMS ESI m/z calcd for [M+Na]^+^ C_30_H_45_NO_17_ 714.2580; found 714.2586.


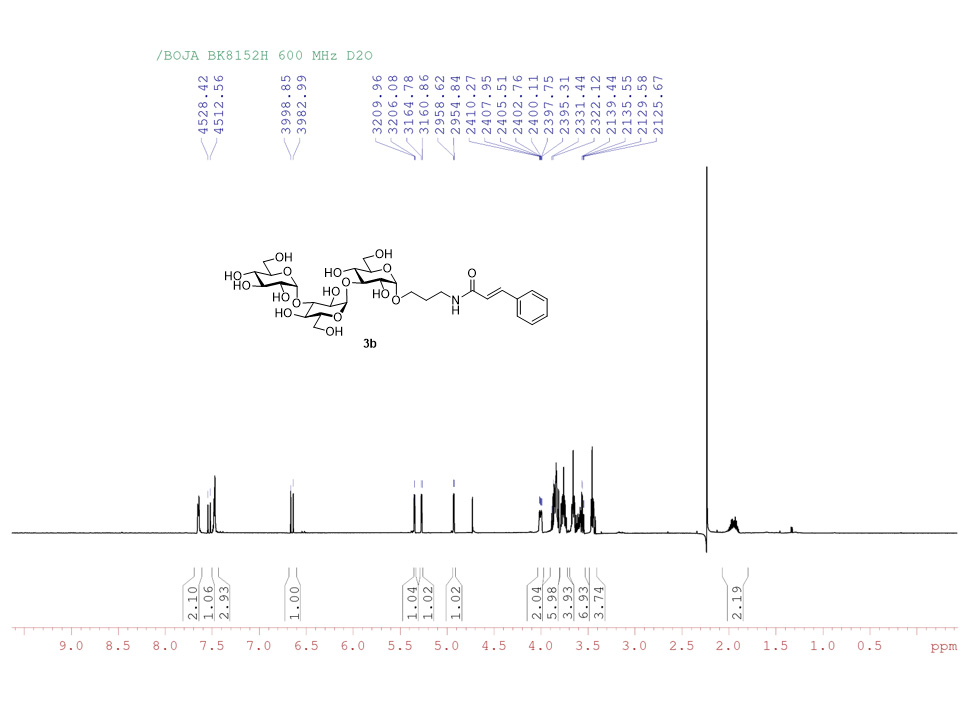


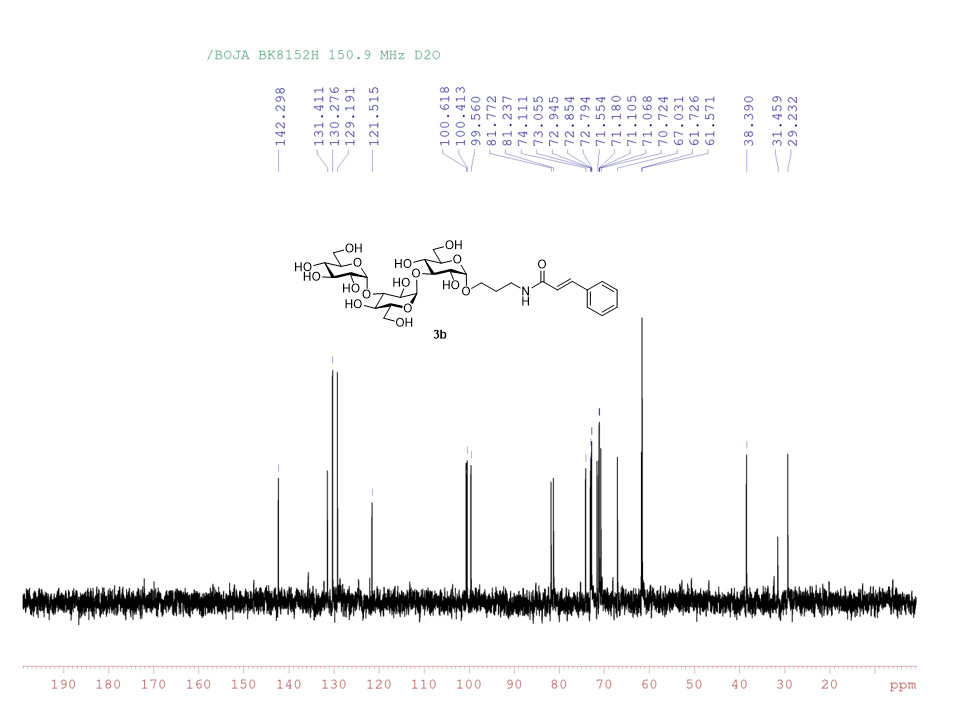


**3-N-*trans*-Cinnamidopropyl α-d-glucopyranosyl-(1→3)-α-d-glucopyranosyl-(1→3)-α-d-glucopyranosyl-(1→3)-α-d-glucopyranoside (4b)** *R*_f_ 0.33 ((MeOH–acetone–CH_2_Cl_2_–H_2_O; 5:15:6:4)–(BuOH–EtOH–H_2_O–NH_3_(15%); 5:5:4:1); 2:1); [α]_D_^25^ = +128.3 (D_2_O, c 0.34). ^1^H NMR (600 MHz, D_2_O): δ_H_ 7.67–7.59 (m, 2H, *o*-Ph), 7.52 (d, *J*_β,α_ 15.7 Hz, CHβ (Cinn)), 7.49–7.43 (m, 3H, *m*-Ph, *p*-Ph), 6.64 (d, *J*_α,β_ 15.7 Hz, CHα (Cinn)), 5.38–5.32 (m, 2H, H-1^C,D^), 5.26 (d, 1H, *J*_1,2_ 3.9 Hz, H-1^B^), 4.91 (d, 1H, *J*_1,2_ 3.9 Hz, H-1^A^), 4.05–3.97 (m, 3H, H-5^B-D^), 3.92–3.69 (m, 13H, H-3^C–A^, H-6^A-D^, H-3^D^, OC*H*_2_CH_2_CH_2_NHCinn(A)), 3.69–3.52 (m, 10H, C-4^A-C^, C-2^A,C^, C-5^A^, OC*H*_2_CH_2_CH_2_NHCinn(B), C-2^D,B^), 3.48–3.39 (m, 3H, OCH_2_CH_2_C*H*_2_NHCinn, H-4^D^), 1.93 (m, 2H, OCH_2_C*H*_2_CH_2_NHCinn). ^13^C NMR (150.9 MHz, CDCl_3_): δ_C_ 142.4 (*C*Hβ (Cinn)), 131.5 (*m*-Ph), 130.3 (*p*-Ph), 129.2 (*o*-Ph), 121.5 (*C*Hα (Cinn)), 100.7 (C-1^D^), 100.6 (C-1^C^), 100.5 (C-1^B^), 99.6 (C-1^A^), 81.7 (C-3^A^), 81.3 (C-3^B^), 81.1 (C-3^C^), 74.1 (C-3^D^), 73.1 (C-2^D^), 73.0, 72.9, 72.8 (C-5^A–D^), 71.6, 71.2, 71.1, (C-2^A–C^, C-4^A–C^), 70.8 (C-4^D^), 67.0 (O*C*H_2_CH_2_CH_2_NHCinn), 61.8, 61.6, 61.5 (C-6^A–D^), 38.4 (OCH_2_CH_2_*C*H_2_NHCinn), 29.2 (OCH_2_*C*H_2_CH_2_NHCinn). HRMS ESI m/z calculated for [M+H]^+^ C_36_H_55_NO_22_ 854.3288; found 854.3285.


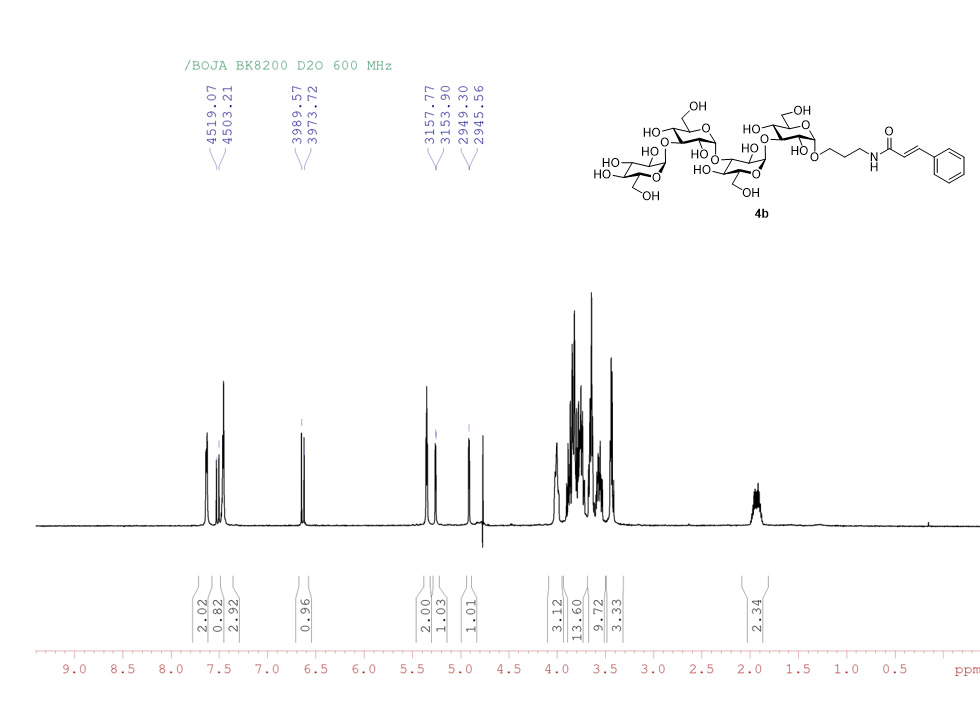


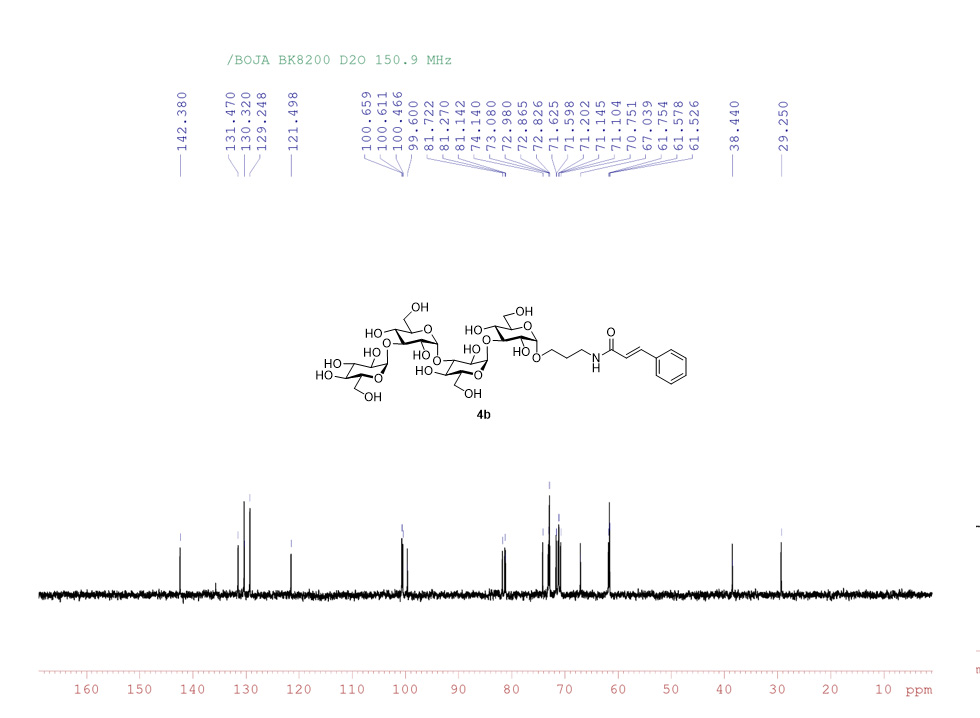


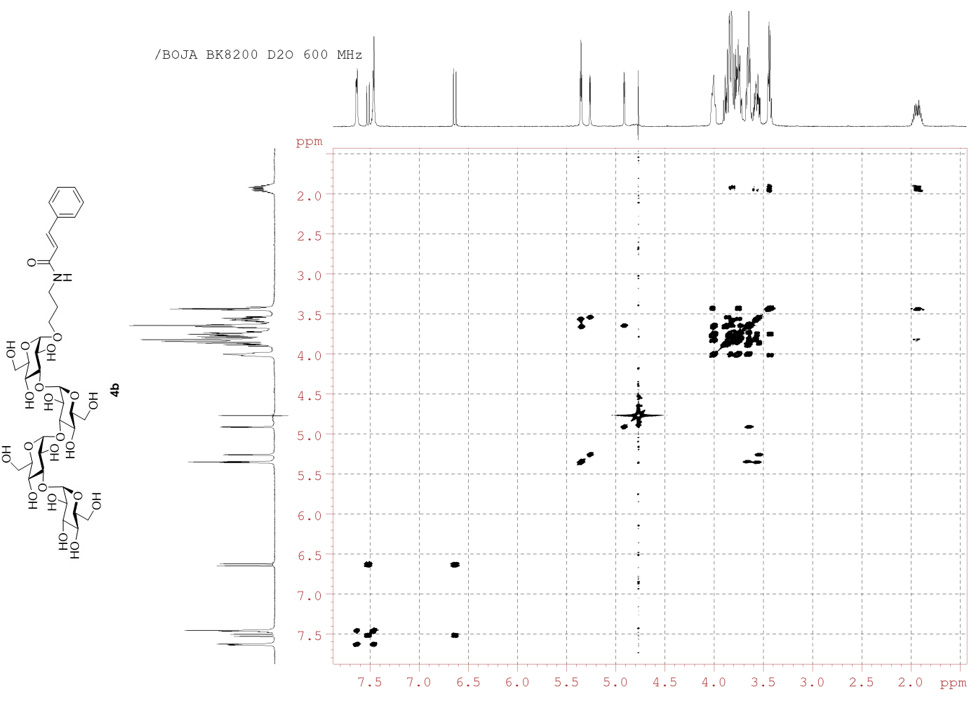


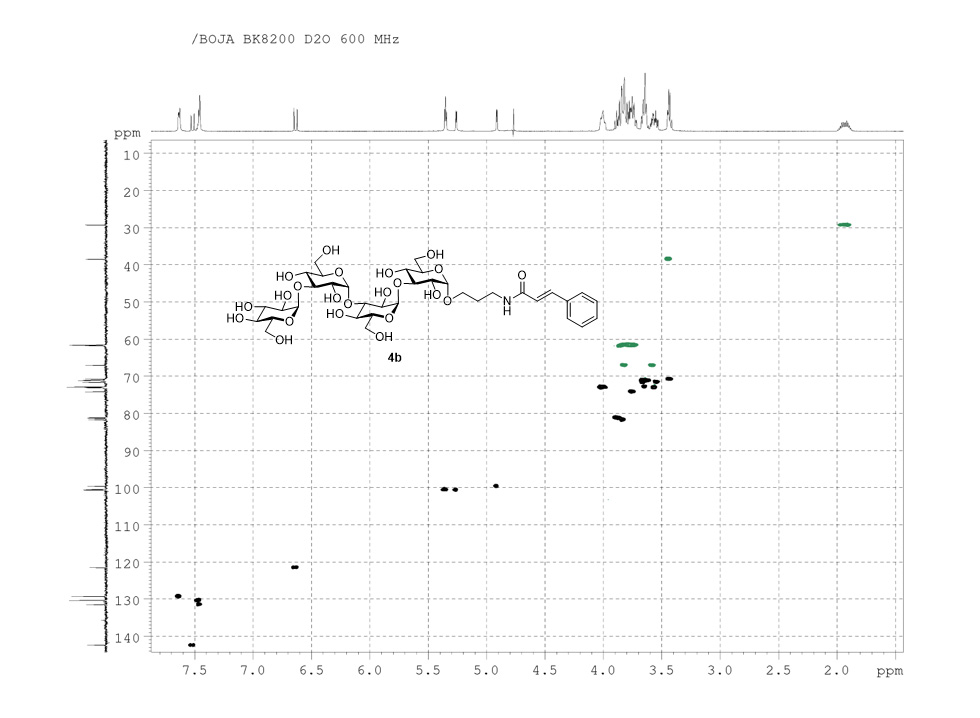


**3-N-*trans*-Cinnamidopropyl α-d-glucopyranosyl-(1→3)-α-d-glucopyranosyl-(1→3)-α-d-glucopyranosyl-(1→3)-α-d-glucopyranosyl-(1→3)-α-d-glucopyranoside (5b)** *R*_f_ 0.44 (BuOH–EtOH–H_2_O–NH_3_ (15%); 5:5:4:1); [α]_D_^25^ = +164.6 (D_2_O, c 0.5). ^1^H NMR (600 MHz, D_2_O): δ_H_ 7.65–7.61 (m, 2H, *o*-Ph), 7.52 (d, *J*_β,α_ 16.1 Hz, CHβ (Cinn)), 7.49–7.44 (m, 3H, *m*-Ph, *p*-Ph), 6.63 (d, *J*_α,β_ 16.1 Hz, CHα (Cinn)), 5.39–5.31 (m, 3H, H-1^C–E^), 5.26 (d, 1H, *J*_1,2_ 3.7 Hz, H-1^B^), 4.92 (d, 1H, *J*_1,2_ 3.7 Hz, H-1^A^), 4.06–3.96 (m, 4H, H-5^B-E^), 3.92–3.52 (m, 28H, H-3^D–A^, H-3^E^, H-6^A-E^, OC*H*_2_CH_2_CH_2_NHCinn(A), H-4^A-D^, H-2^A,C,D^, H-5^A^, OC*H*_2_CH_2_CH_2_NHCinn(B), H-2^E,B^), 3.46–3.40 (m, 3H, OCH_2_CH_2_C*H*_2_NHCinn, H-4^D^), 1.93 (m, 2H, OCH_2_C*H*_2_CH_2_NHCinn). ^13^C NMR (150.9 MHz, CDCl_3_): δ_C_ 142.3 (*C*Hβ (Cinn)), 131.4 (*m*-Ph), 130.3 (*p*-Ph), 129.2 (*o*-Ph), 121.7 (*C*Hα (Cinn)), 100.6, 100.5 (C-1^B-E^), 99.6 (C-1^A^), 82.0 (C-3^A^), 81.4 (C-3^B–E^), 74.2 (C-3^E^), 73.1, 73.0, 72.9 (C-5^A-E^, C-2^E^), 71.7, 71.0 (C-2^B^, C-2^A,C–D^, C-4^A-D^), 70.8 (C-4^E^), 67.2 (O*C*H_2_CH_2_CH_2_NHCinn), 61.8, 61.6 (C-6^A–E^), 38.4 (OCH_2_CH_2_*C*H_2_NHCinn), 29.3 (OCH_2_*C*H_2_CH_2_NHCinn). HRMS ESI m/z calculated for [M+H]^+^ C_42_H_65_NO_27_ 1016.3817; found 1016.3806.


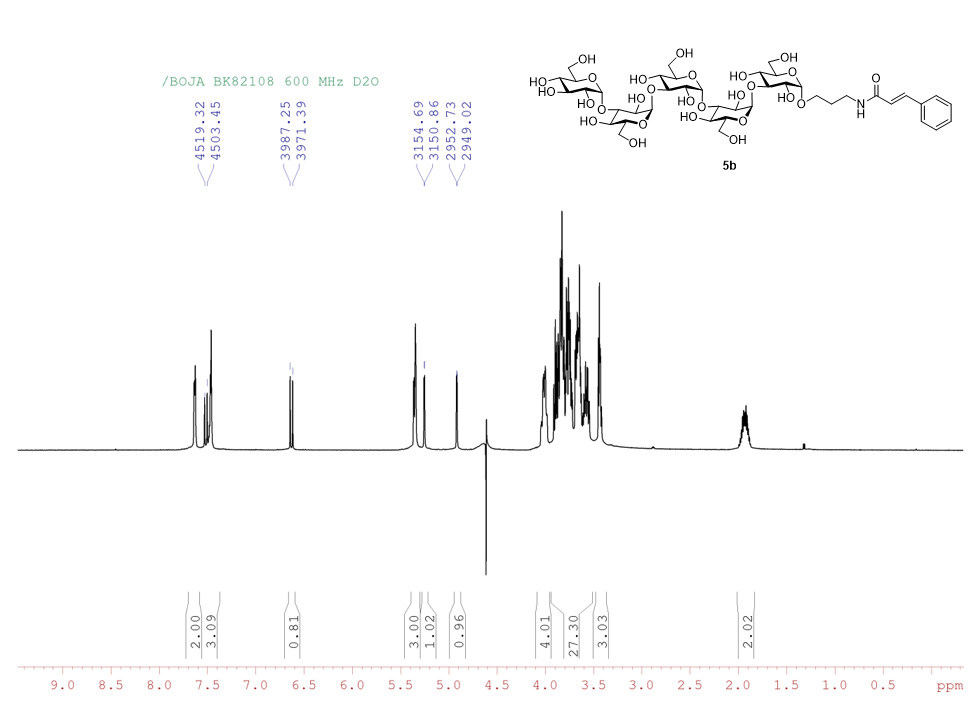


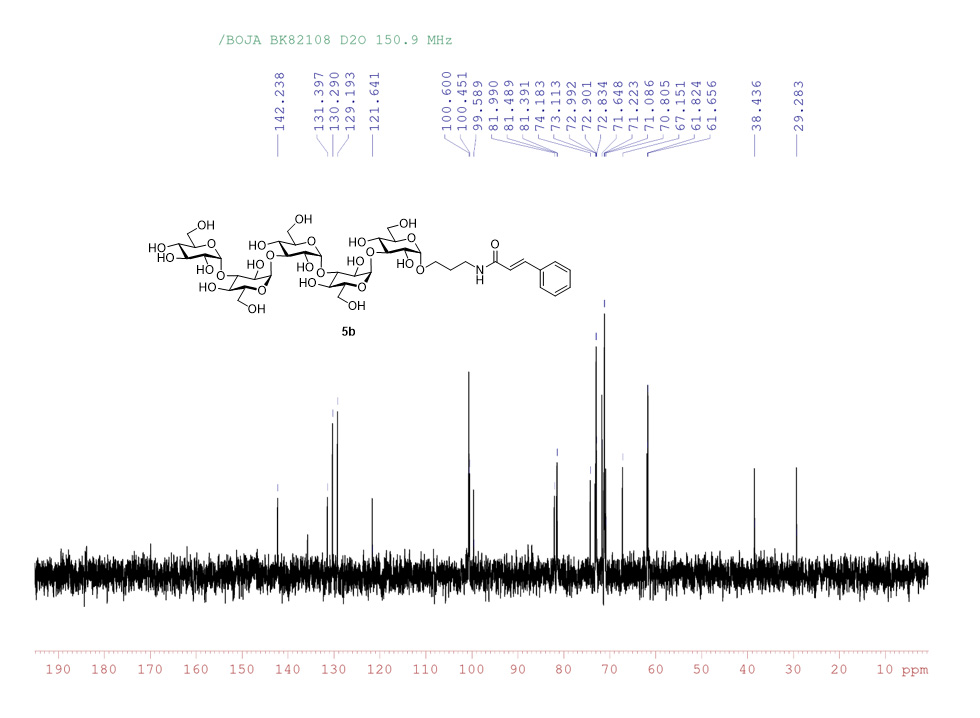


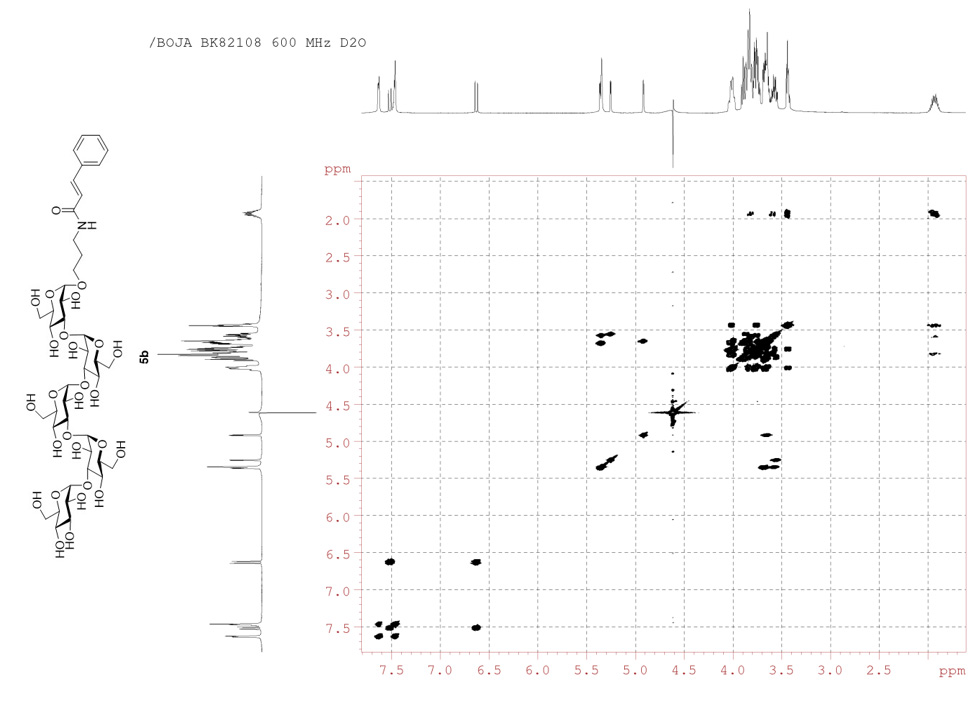


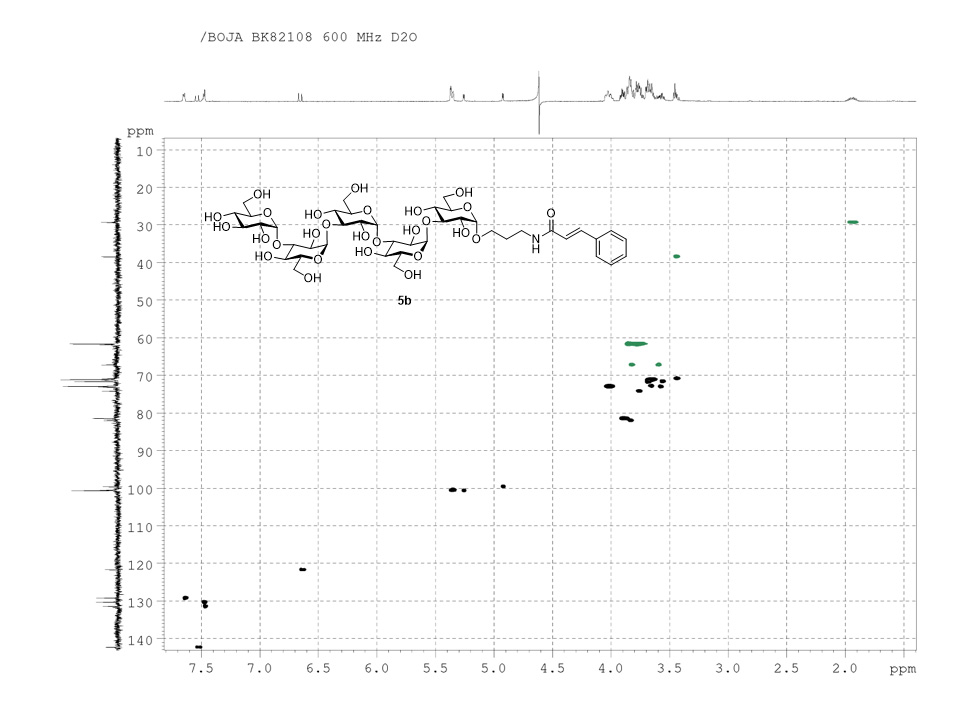


**3-N-*trans*-Cinnamidopropyl α-D-glucopyranosyl-(1→3)-α-d-glucopyranosyl-(1→3)-α-d-glucopyranosyl-(1→3)-α-d-glucopyranosyl-(1→3)-α-d-glucopyranosyl-(1→3)-α-d-glucopyranosyl-(1→3)-α-d-glucopyranoside (6b)** *R*_f_ 0.70 (BuOH–EtOH–H_2_O–NH_3_ (15%); 0.5:1:0.8:0.5); [α]_D_^21^ = +113.4 (D_2_O, c 0.22). ^1^H NMR (600 MHz, D_2_O): δ_H_ 7.68–7.62 (m, 2H, *o*-Ph), 7.54 (d, 1H, *J*_β,α_ 15.5 Hz, CHβ (Cinn)), 7.50–7.46 (m, 3H, *m*-Ph, *p*-Ph), 6.65 (d, *J*_α,β_ 15.6 Hz, CHα (Cinn)), 5.38–5.33 (m, 5H, H-1^C–G^), 5.26 (d, 1H, *J*_1,2_ 4.0 Hz, H-1^B^), 4.92 (d, 1H, *J*_1,2_ 3.9 Hz, H-1^C^), 4.06–3.97 (m, 6H, H-5^B-G^), 3.94–3.53 (m, 38H, H-3^F–A^, H-3^G^, H-6^A-F^, OC*H*_2_CH_2_CH_2_NHCinn(A), C-4^A-F^, C-2^A,C-F^, C-5^A^, OC*H*_2_CH_2_CH_2_NHCinn(B), C-2^G,B^), 3.47–3.41 (m, 3H, OCH_2_CH_2_C*H*_2_NHCinn, H-4^D^), 1.94 (m, 2H, OCH_2_C*H*_2_CH_2_NHCinn). ^13^C NMR (150.9 MHz, CDCl_3_): δ_C_ 142.3 (*C*Hβ (Cinn)), 131.4 (*m*-Ph), 130.3 (*p*-Ph), 129.2 (*o*-Ph), 121.7 (*C*Hα (Cinn)), 100.6, 100.5 (C-1^B-G^), 99.6 (C-1^A^), 82.0 (C-3^A^), 81.4 (C-3^B-F^), 74.2 (C-3^G^), 73.1, 73.0, 72.9 (C-5^B-G^, C-5^A^, C-2^G^), 71.7, 71.1 (C-2^B^, C-2^A,C–F^, C-4^A-F^), 70.8 (C-4^G^), 67.2 (O*C*H_2_CH_2_CH_2_NHCinn), 61.8, 61.6 (C-6^A–G^), 38.4 (OCH_2_CH_2_*C*H_2_NHCinn), 29.3 (OCH_2_*C*H_2_CH_2_NHCinn). HRMS ESI m/z calculated for [M+Na]^+^ C_54_H_85_NO_37_ 1362.4693; found 1362.4688.


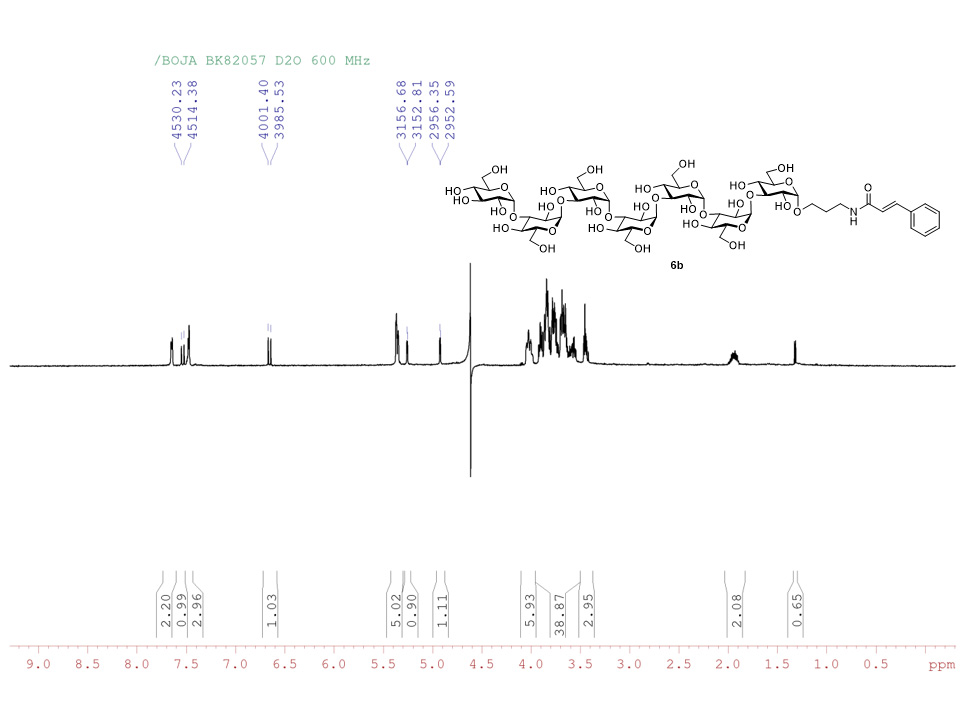


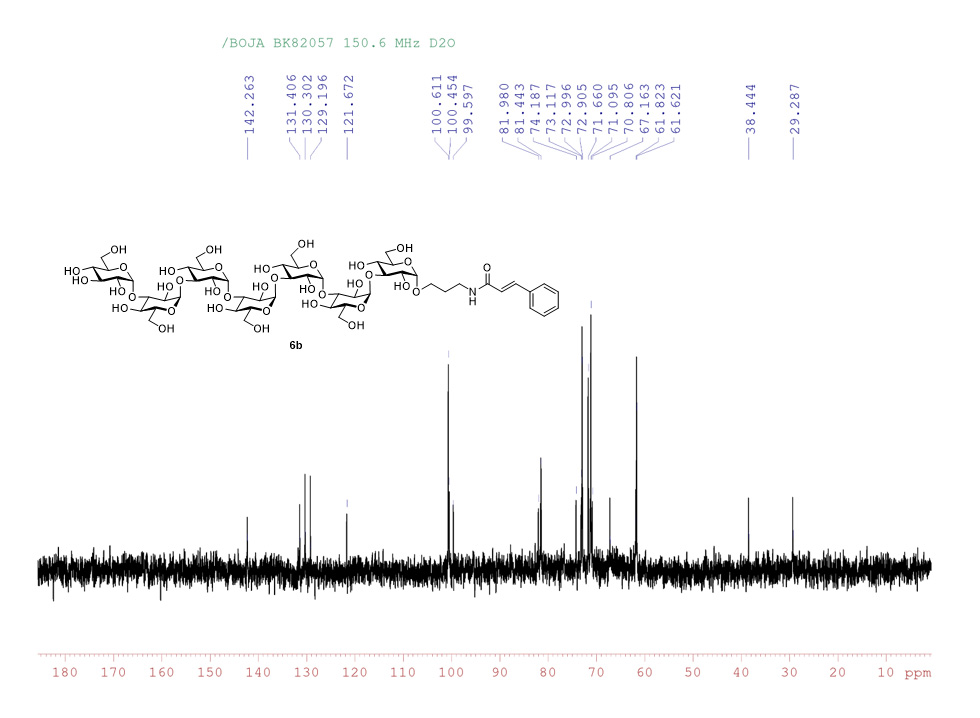


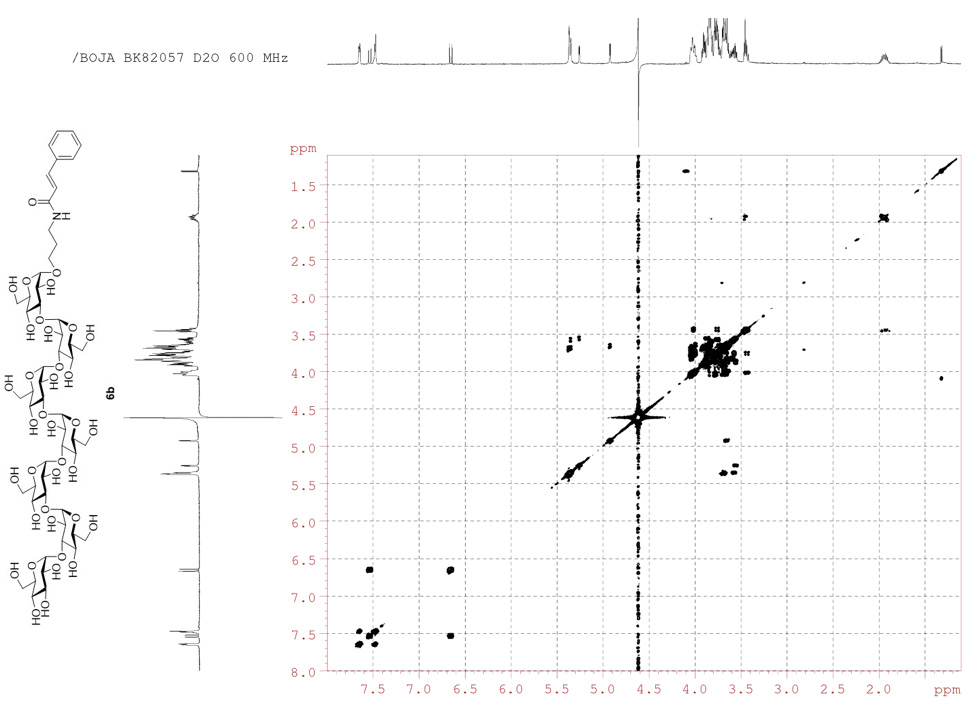


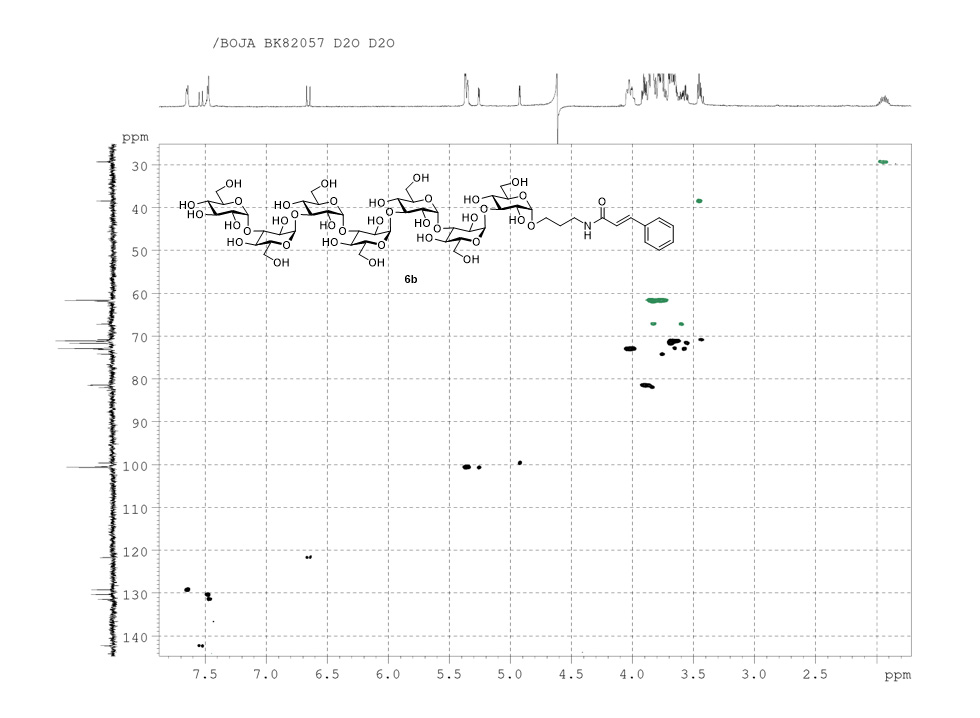


**3-N-*trans*-Cinnamidopropyl 2-deoxy-2-acetamido-β-d-glucopyranosyl-(1→3)-α-d-glucopyranosyl-(1→3)-α-d-glucopyranosyl-(1→3)-α-d-glucopyranosyl-(1→3)-α-d-glucopyranosyl-(1→3)-α-d-glucopyranoside (7b)** *R*_f_ 0.78 (BuOH–EtOH–H_2_O–NH_3_ (15%); 0.5:1:0.8:0.5); [α]_D_^31^ = +115.0 (D_2_O, c 0.64). ^1^H NMR (600 MHz, D_2_O): δ_H_ 7.65–7.60 (m, 2H, *o*-Ph), 7.51 (d, *J*_β,α_ 15.9 Hz, CHβ (Cinn)), 7.48–7.42 (m, 3H, *m*-Ph, *p*-Ph), 6.63 (d, *J*_α,β_ 15.9 Hz, CHα (Cinn)), 5.35 (d, 1H, , *J*_1,2_ 3.6 Hz, H-1^B,C or D^), 5.32 (d, 1H, , *J*_1,2_ 3.9 Hz, H-1^B,C or D^), 5.30 (d, 1H, , *J*_1,2_ 3.7 Hz, H-1^B,C or D^), 5.23 (d, 1H, , *J*_1,2_ 3.7 Hz, H-1^E^), 4.90 (d, 1H, , *J*_1,2_ 3.6 Hz, H-1^A^), 4.73 (d, 1H, , *J*_1,2_ 8.7 Hz, H-1^F^), 4.04–3.95 (m, 4H, H-5^B-E^), 3.93–3.69 (m, 19H, H-6^A-F^, H-3^E–A^, OC*H*_2_CH_2_CH_2_NHCinn(A), H-2^F^), 3.67–3.51 (m, 12H, H-4^A–D^, H-2^A–D^, H-5^A^, OC*H*_2_CH_2_CH_2_NHCinnB, H-3^F^, H-2^E^), 3.51–3.41 (m, 5H, OCH_2_CH_2_C*H*_2_NHCinn, H-4^E^, H-4^F^, H-5^F^), 2.03 (s, 3H, CH_3_ (AcNH)), 1.92 (m, 2H, OCH_2_C*H*_2_CH_2_NHCinn). ^13^C NMR (150.9 MHz, CDCl_3_): δ_C_ 142.8 (*C*Hβ (Cinn)), 131.9 (*m*-Ph), 130.8 (*p*-Ph), 129.7 (*o*-Ph), 122.2 (*C*Hα (Cinn)), 103.5 (C-1^F^), 101.2 (C-1^E^), 101.1 (C-1^C,D^), 101.0 (C-1^B^), 100.1 (C-1^A^), 83.9 (C-3^E^), 82.5 (C-3^A^), 82.0, 81.9, 81.7 (C-3^B-D^), 77.6 (C-5^F^), 75.5 (C-3^F^), 73.4, 73.3, 73.3, 73.2 (C-5^A-E^, C-2^B^), 72.2, 72.1, 71.7, 71.6 (C-4^A–D,F^, C-2^A,C-E^), 69.8 (C-4^E^), 67.7 (O*C*H_2_CH_2_CH_2_NHCinn), 62.5, 62.3, 62.2, 62.1 (C-6^A–F^), 57.6 (C-2^F^), 38.9 (OCH_2_CH_2_*C*H_2_NHCinn), 29.8 (OCH_2_*C*H_2_CH_2_NHCinn), 24.0 (CH_3_ (AcNH)). HRMS ESI m/z calculated for [M+H]^+^ C_50_H_78_N_2_O_32_ 1219.4610; found 1219.4622.


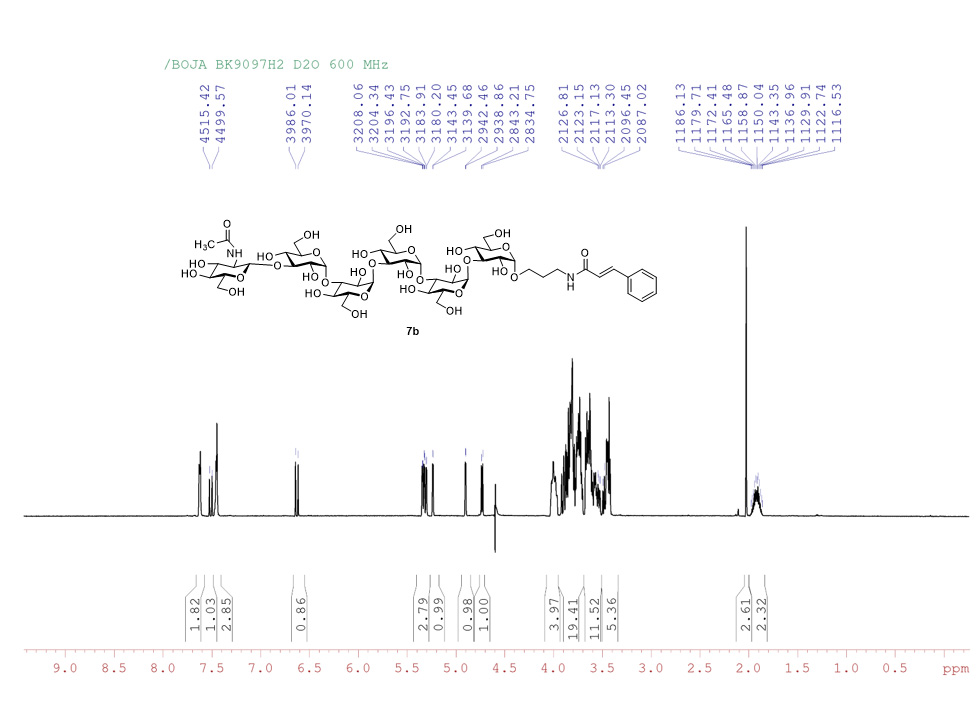


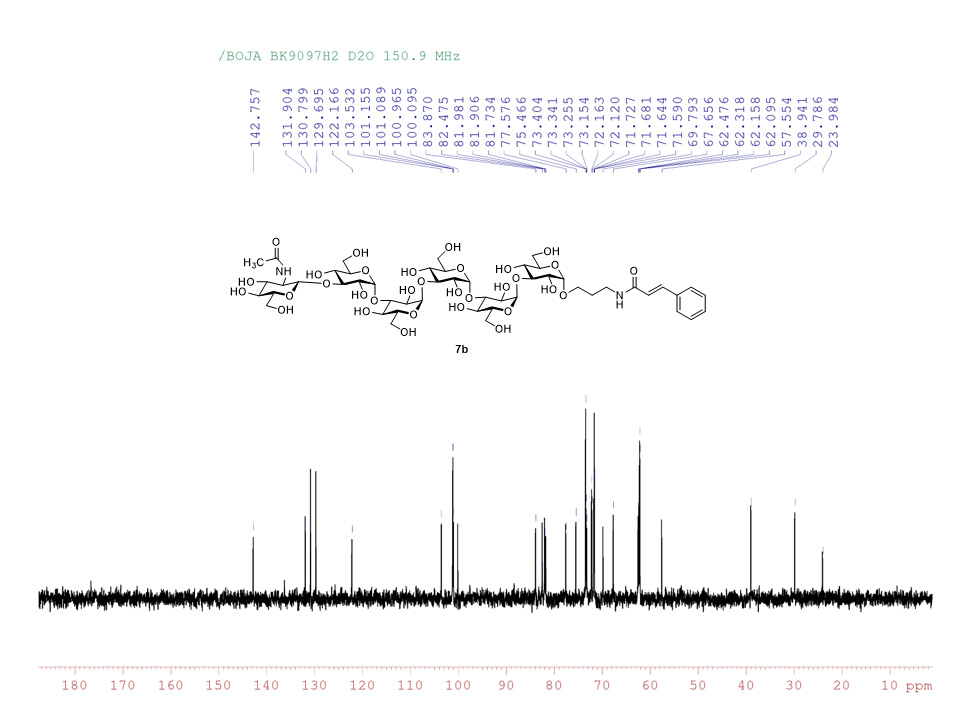


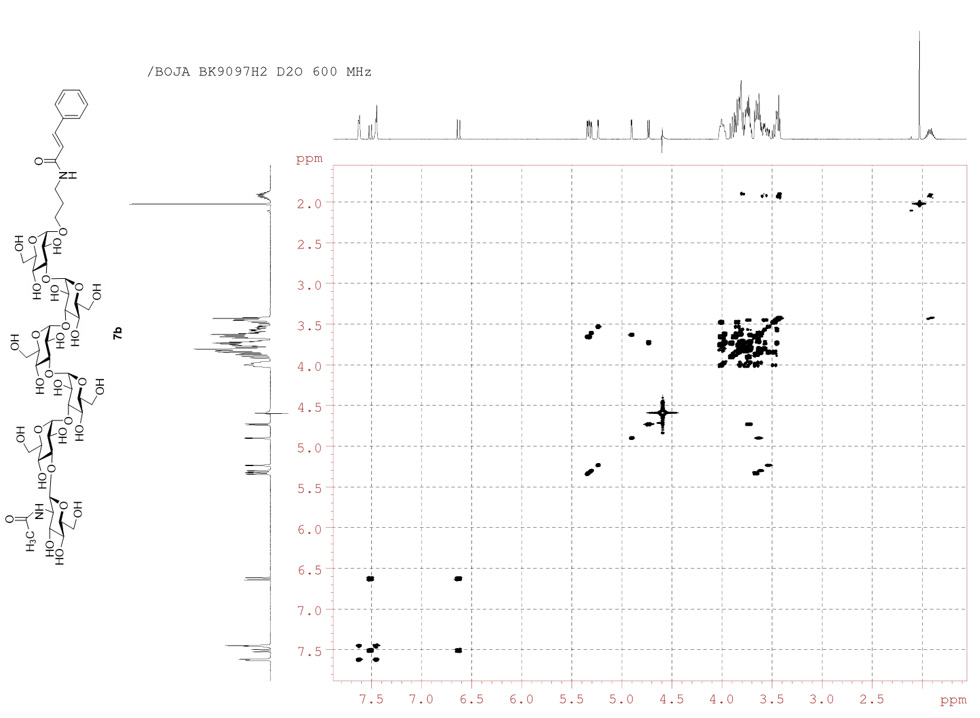


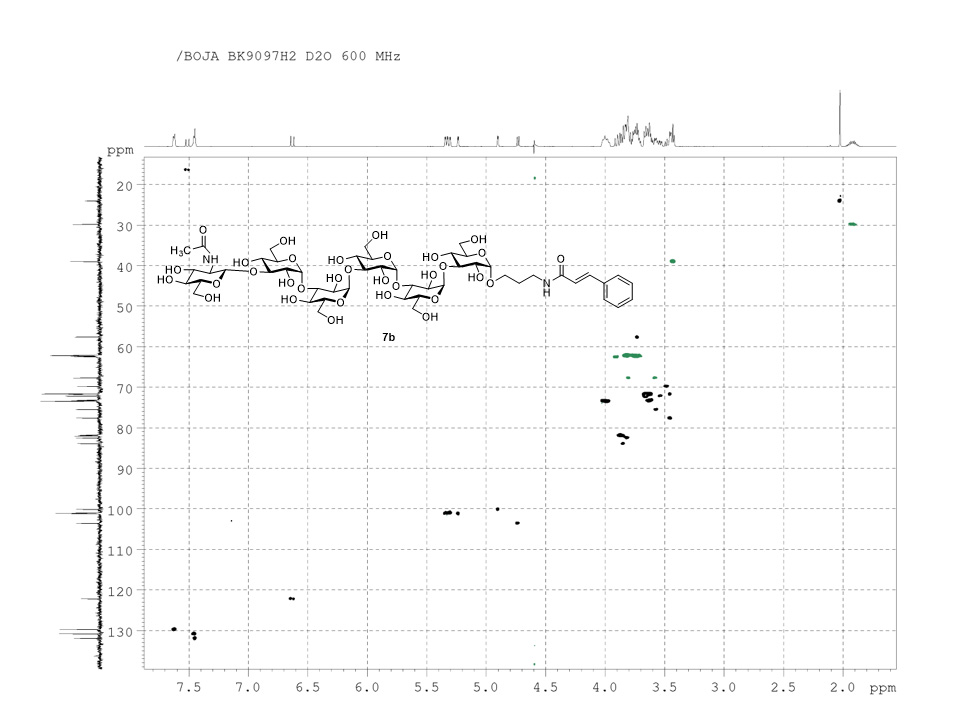


**A**

atgttgggcgttttccgccgcctcaggctcggcgcccttgccgccgcagctctgtcttctctcggcagtgccgctcccgccaatgttgctattcggtctctcgaggaacgtgcttcttctgctgaccgtctcgtattctgtcacttcatgattggtatcgttggtgatcgtggcagctcggcagactatgatgacgatatgcaacgtgccaaagccgctggcattgacgccttcgccctgaacatcggcgttgacggctataccgaccagcagctcggatatgcctatgactctgccgatcgtaatggcatgaaagtcttcatctcattcgatttcaattggtggagccccggcaatgcagttggtgttggccagaagattgcgcagtacgccaaccgccctgcccagctgtatgtcgacaaccgaccattcgcctcttccttcgccggtgacggtctagatgtaaatgcgttgcgctctgctgcaggctccaacgtttactttgttcccaacttccaccctggccaatcttctccctcaaacattgatggtgcccttaactggatggcgtagtcgcaacttagagtcgagagcaggggagcaatactgatgtctttatagctgggataatgatggaaacaacaaggcacccaagccgggccagactgtcacggtggcagacggtgacaacgcttacaagaattggttgggtggcaagccttacctagcgcctgtctcaccttggttttttacccatttcggccccgaagtttcatactccaagaactgggtcttcccaggtggtcctctgatctacaaccggtggcaacaggtcttgcagcagggcttccccatggttgagatcgttacctggaatgactacggcgagtctcactacgtcggtccactaaagtctaagcatttcgatgatggcaactccaaatgggtcaatgatatgccccatgatggattcctggatctttcaaagccgtttattgctgcgtataagaacagggataccgacatctccaagtatgttcaaaatgagcagcttgtttactggtaccgccgcaacttgaaggcattggactgcgacgccaccgacaccacctctaaccgtccagctaataacggaagtggtaattactttatgggacgtcctgatggttggcaaactatggatgacaccgtttatgttgccgcacttctcaagactgccggtagcgtcactgtcacgtctggcggcaccactcaaacgttccaggccaacgccggagccaacctcttccaaatcccagccagcatcggccagcaaaaattcgctctaactcgcaatggtcagaccgtctttagtggaacctcattgatggatatcaccaacgtttgctcttgcggtatctacaacttcaacccatatgttggcaccattcctgccggctttgacgaccctctacaggctgatggtcttttctctttgaccatcggattgcatgtcacaacttgtcaggccaagccatctcttggaaccaaccctcctgtcacttccggccctgtgtcctcgcttccagcttcctccaccacccgcgcatcctcgccgcctcctgtttcttccactcgtgtctcttctccccctgtctcttcccctccagtttctcgcacctcttccactccccctccagcgagcagcacgccgccatcgggtcaggtttgcgttgccggtaccgttgctgacggcgagtctggcaactacatcggcctgtgccaattcagctgcaacgtccccataacccttacttgcttccttaactaatccttcatagtacggttactgcccaccaggaccgtgtaagtgcaccgcctttggtgctcccatcaacccaccggcaagcaatggccgcaacggctgccctctgccgggagaaggcgatggttatctgggcctgtgcagtttcagttgtaaccataattactgccccccaacggcatgtcaatactgctaa

**B**

MLGVFRRLRLGALAAAALSSLGSAAPANVAIRSLEERASSADRLVFCHFMIGIVGDRGSSADYDDDMQRAKAAGIDAFALNIGVDGYTDQQLGYAYDSADRNGMKVFISFDFNWWSPGNAVGVGQKIAQYANRPAQLYVDNRPFASSFAGDGLDVNALRSAAGSNVYFVPNFHPGQSSPSNIDGALNWMAWDNDGNNKAPKPGQTVTVADGDNAYKNWLGGKPYLAPVSPWFFTHFGPEVSYSKNWVFPGGPLIYNRWQQVLQQGFPMVEIVTWNDYGESHYVGPLKSKHFDDGNSKWVNDMPHDGFLDLSKPFIAAYKNRDTDISKYVQNEQLVYWYRRNLKALDCDATDTTSNRPANNGSGNYFMGRPDGWQTMDDTVYVAALLKTAGSVTVTSGGTTQTFQANAGANLFQIPASIGQQKFALTRNGQTVFSGTSLMDITNVCSCGIYNFNPYVGTIPAGFDDPLQADGLFSLTIGLHVTTCQAKPSLGTNPPVTSGPVSSLPASSTTRASSPPPVSSTRVSSPPVSSPPVSRTSSTPPPASSTPPSGQVCVAGTVADGESGNYIGLCQFSCNYGYCPPGPCKCTAFGAPINPPASNGRNGCPLPGEGDGYLGLCSFSCNHNYCPPTACQYC

**FIG S3** Nucleotide sequence for *T. harzianum* MutA (A), translated amino acid sequence for *T. harzianum* MutA (B). Matching tryptic peptides identified by MALDI-TOF MS are shown in red and blue color.

**FIG S4** The SDS-PAGE of the crude enzyme preparations (culture filtrates) produced by recombinant strains M18 (*1*), M22 (*2*), M25 (*3*) and M37 (*4*) of *P. verruculosum*, carrying the *mutA* gene, and the control sample (*5*) produced by *P. verruculosum* B537 (ΔniaD) host strain.

**FIG S5** SDS-PAGE of purified MutA.

**FIG S6** MALDI-TOF mass spectrum of the target MutA recorded on an UltrafleXtreme II instrument. Designations: a.u., arbitrary units.


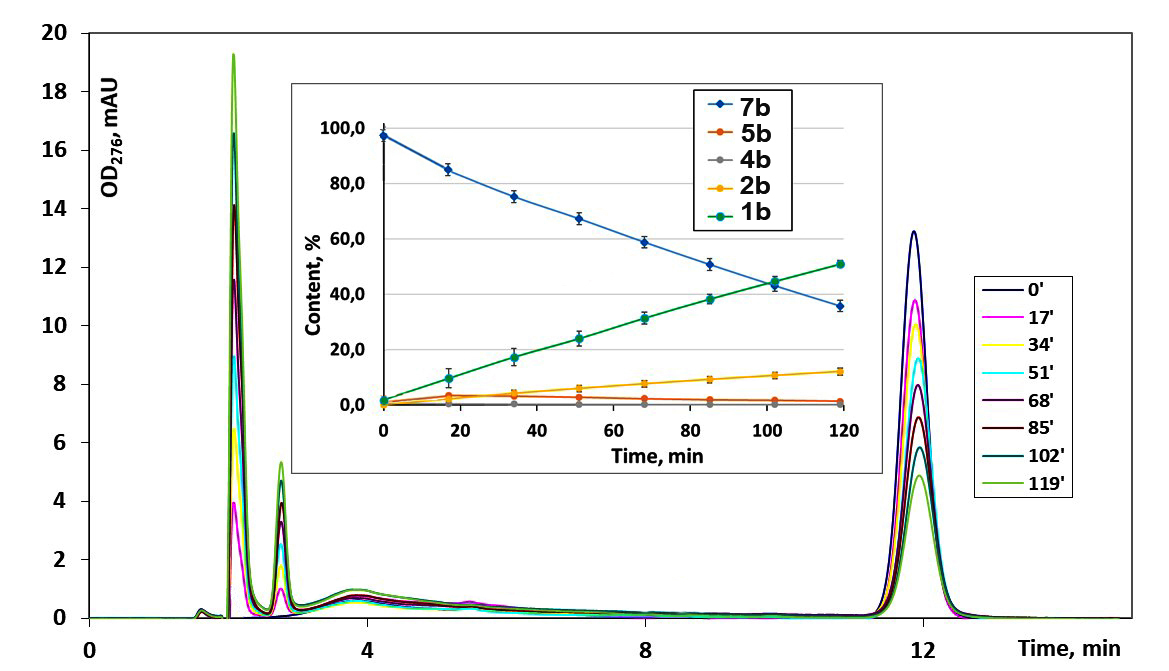


**FIG S7** HPLC profile and kinetics of **7b** hydrolysis. Designations: OD_276_, optical density at 276 nm.

**Table S1**. Activity (U/ml) in the fermentation medium during the growing of the recombinant strains of *P. verruculosum*, carrying the *mutA* gene, and of the host strain *P. verruculosum* B537 (ΔniaD)

| **Clone number** |  |  |  |  |  |
| --- | --- | --- | --- | --- | --- |
|  | **48h** | **72h** | **96h** | **120h** | **144h** |
| М18 | 5.0±0.4 | 51±1.5 | 80±6 | 104±4 | 132±7.5 |
| М22 | 20±1.5 | 140±11 | 221±1.4 | 272±5 | **310**±8 |
| М25 | 10±1.2 | 102±4 | 180±10 | 211±4 | 250±10 |
| М37 | 10±1.0 | 93±4 | 141±8 | 183±6 | 210±12 |
| PV-control | 0.1±0.02 | 0.5±0.01 | 1.5±0.09 | 2.0±0.11 | 3.2±0.09 |
